# GLP-1/GLP-1R signalling in infectious diseases: basic biology, immunometabolic mechanisms, and therapeutic translation

**DOI:** 10.1186/s43556-026-00645-8

**Published:** 2026-11-11

**Authors:** Xue Yue, Haifeng Fu, Hang Qian, Binqiang Zhang, Hao Xu, Dongfeng Li, Jishun Chen, Xinwen Min, Handong Yang, Wenwen Wu, Zhixin Liu, Aihua Mei, Jun Chen

**Affiliations:** 1 Sinopharm Dongfeng General Hospital (Hubei Clinical Research Center of Hypertension), Hubei Key Laboratory of Wudang Local Chinese Medicine Research, Hubei University of Medicine, Shiyan, Hubei, 442000 China; 2 Shiyan Key Laboratory of Virology, Hubei University of Medicine, Shiyan, China; 3 School of Public Health, Hubei University of Medicine, Shiyan, Hubei, 442000 China

**Keywords:** GLP-1, GLP-1 receptor, GLP-1 receptor agonists, Infectious diseases, Immunometabolism, Host defence

## Abstract

Infectious diseases remain a major global health burden, and metabolic disorders such as diabetes and obesity further increase susceptibility to infection and adverse outcomes. These intersecting metabolic and infectious risks have prompted growing interest in the glucagon-like peptide-1 (GLP-1)/GLP-1 receptor (GLP-1R) axis beyond its established role in glycaemic regulation. GLP-1 receptor agonists (GLP-1RAs) modulate systemic metabolism, neuroimmune signalling, vascular and epithelial homeostasis, and inflammatory responses. Whether their infection-related effects arise from direct immune-cell GLP-1R signalling, improved pathogen control, or indirect host protection remains unresolved. This narrative review critically evaluates GLP-1/GLP-1R signalling in infection. It distinguishes live-pathogen models from endotoxin and sterile-injury models, host resistance from host tolerance, and direct receptor-dependent actions from indirect systemic effects. It also differentiates chronic pre-infection exposure from treatment initiated after infection onset. Across selected preclinical models, GLP-1RAs more consistently attenuate inflammation and tissue injury while preserving organ function, whereas their effects on pathogen burden and clearance remain variable. Receptor-specific evidence for immune-cell-intrinsic GLP-1R signalling remains limited, and many reported immune phenotypes may instead reflect metabolic improvement, neuroimmune regulation, or barrier protection. Human evidence is derived mainly from observational studies and secondary analyses of chronic GLP-1RA exposure, with limited evidence from trials initiating treatment during acute infection. Future translation will require cell-specific receptor validation, live-pathogen models with post-infection treatment, parallel assessment of pathogen control and host protection, clinically relevant standard-of-care comparators, and infection-specific safety evaluation. GLP-1RAs are therefore best regarded as candidate host-directed adjuncts. Current evidence does not establish them as direct anti-infective therapies.

## Introduction

Infectious diseases remain a major cause of global mortality and disability, disproportionately affecting children, older adults, and individuals with underlying disorders [1]. In 2023, lower respiratory tract infections alone caused approximately 2.5 million deaths and 98.7 million disability-adjusted life years worldwide [1]. Antimicrobial resistance further intensifies this burden, accounting for an estimated 4.71 million associated deaths in 2021, including 1.14 million directly attributable to resistant bacterial infections [2]. Infection outcomes are determined not only by pathogen burden and treatment failure but also by dysregulated inflammation, metabolic stress, barrier dysfunction, endothelial and microcirculatory injury, and progressive organ dysfunction [3]. Recognition of these host-mediated components of disease has increased interest in host-directed therapies that complement antimicrobial treatment by limiting immunopathology and preserving tissue and organ function [4].

Glucagon-like peptide-1 (GLP-1), produced mainly by intestinal L cells and preproglucagon-expressing brainstem neurons, acts through the GLP-1 receptor (GLP-1R) and canonical Gαs–cAMP–PKA/Epac signalling to regulate glucose-dependent insulin secretion, glucagon release, gastric emptying, appetite, and energy intake [5]. GLP-1 receptor agonists (GLP-1RAs), widely used for type 2 diabetes and obesity, also provide cardiovascular and renal benefits in selected high-risk populations [6, 7]. Beyond these established metabolic actions, GLP-1/GLP-1R signalling has been linked to immunometabolic regulation, neuroimmune communication, and epithelial, endothelial, and tissue homeostasis [8, 9]. Functional GLP-1R expression in many immune-cell populations remains uncertain [8, 10]. This uncertainty limits direct attribution of treatment-associated immune changes to immune-cell GLP-1R signalling. Such changes may also reflect systemic metabolic improvement, central neuronal regulation, vascular or barrier protection, or remodelling of the local tissue microenvironment [8, 9]. Infection-related studies span endotoxin-based and other non-live-pathogen models, live-pathogen experiments, and observational clinical analyses [11–14].

This narrative review critically evaluates the biological basis, mechanistic plausibility, and infection-related evidence for GLP-1/GLP-1R signalling. We consider GLP-1 biology, GLP-1R distribution and receptor-validation challenges, canonical signalling, immunometabolic and neuroimmune regulation, immune-cell responses, barrier protection, autophagy, and mitochondrial homeostasis in relation to host resistance and host tolerance. Preclinical and clinical findings are evaluated across respiratory, systemic bacterial, gastrointestinal, hepatic, fungal, and parasitic infections, as well as in metabolically high-risk hosts. The analysis distinguishes sterile from live-pathogen models, prophylactic from post-diagnosis treatment, and direct receptor-dependent actions from indirect systemic effects. We also address agent-specific pharmacology, safety, combination strategies, and priorities for infection-focused clinical translation.

## Basic biology of GLP-1/GLP-1R

### Sources, secretion, and degradation of GLP-1

Glucagon-like peptide-1 (GLP-1) is generated by tissue-specific processing of *Gcg*-encoded proglucagon and classically promotes glucose-dependent insulin secretion, suppresses glucagon release, slows gastric emptying, and regulates appetite [5]. GLP-1 is increasingly viewed as part of an integrated GLP-1/GLP-1R axis that links nutrient sensing, neuroendocrine control, immunometabolism, and tissue protection, with potential relevance to infectious diseases [5].

In the periphery, GLP-1 is produced mainly by enteroendocrine L cells in the ileum and colon. These cells sense luminal glucose, fatty acids, and amino acids through nutrient transporters, ion channels, and Ca2⁺-dependent pathways [5, 15]. Microbiota-derived short-chain fatty acids (SCFAs) also stimulate GLP-1 secretion via receptors including FFAR2 and FFAR3 [16, 17]. Gut microbiota regulates the postprandial GLP-1 response via ileal bile acid–TGR5 signalling [18]. GLP-1RAs may also alter gut microbial composition through changes in intestinal motility, bile acid metabolism, and energy balance [19, 20]. These findings support a bidirectional relationship between intestinal GLP-1 signalling and the gut microbiota, although most evidence comes from metabolic settings [18–20].

GLP-1 is also synthesised within the central nervous system, predominantly by brainstem preproglucagon (PPG) neurons located in and around the nucleus tractus solitarius [21]. These neurons project widely to the hypothalamus, limbic structures, and other regions involved in feeding, energy balance, stress responses, and autonomic regulation, where GLP-1 acts as a neurotransmitter or neuromodulator [21, 22]. Unlike circulating GLP-1, brain-derived GLP-1 acts mainly within local neural circuits. Central GLP-1R activation in these circuits may engage neuroimmune reflexes that limit systemic inflammation and reduce lung injury in polymicrobial sepsis [9, 21].

Once secreted, bioactive GLP-1 is present mainly as GLP-1(7–36) amide but is rapidly inactivated through N-terminal cleavage by dipeptidyl peptidase-4 (DPP-4), yielding GLP-1(9–36) and related metabolites [5, 23]. This short biological persistence limits the insulinotropic activity of endogenous GLP-1 and underpins the development of both degradation-resistant GLP-1RAs and DPP-4 inhibitors [5]. Although GLP-1(9–36) was historically considered inactive or weakly active, evidence indicates that it may retain non-canonical biological activity, including suppression of glucagon secretion through mechanisms not entirely dependent on classical GLP-1R signalling [23]. DPP-4 regulates the persistence of endogenous GLP-1. Its membrane-associated form, CD26, also has independent immunoregulatory functions [24, 25].

GLP-1 is therefore better viewed as part of a broader signalling network that integrates enteroendocrine secretion, central neural control, DPP-4-dependent processing, and bioactive metabolites. Through these pathways, GLP-1 may influence host defence via microbiota–mucosal barrier interactions, neuroimmune reflexes, and non-canonical metabolic pathways [5, 9, 23].

### GLP-1R distribution and functional validation

GLP-1R is a class B G protein-coupled receptor whose biological actions are determined by its tissue-specific expression, precise cellular localisation, and the methodological reliability of receptor detection [5, 26, 27]. The most firmly established site of GLP-1R expression is the pancreatic β cell, in which receptor activation mediates canonical metabolic responses, particularly glucose-dependent insulin secretion [26, 28]. Outside the islets, GLP-1R or GLP-1R-associated signalling has been described in the gastrointestinal tract, lungs, cardiovascular system, kidneys, and central nervous system [26, 27]. Receptor abundance, cellular assignment, and functional relevance, however, differ substantially by species, anatomical site, and analytical method [26, 27]. In infection, GLP-1R biology should therefore be interpreted as a multilevel system encompassing β-cell-driven metabolic improvement, central neuroimmune regulation, peripheral barrier and parenchymal organ protection, and possible direct receptor signalling in selected immune-cell subsets.

Within the gastrointestinal tract, GLP-1R-associated signalling involves enteric neural, endocrine, and selected epithelial compartments that contribute to gastrointestinal motility, nutrient sensing, and mucosal homeostasis [27, 29]. Because barrier failure, dysbiosis, and bacterial translocation amplify infectious enteritis, gut-derived sepsis, and multiorgan injury, intestinal GLP-1R signalling may indirectly modify host responses through effects on motility, mucosal integrity, local inflammation, and microbiota–metabolite interactions [30, 31]. However, most supporting data arise from metabolic disease, inflammatory bowel disease, or sterile barrier-injury models and therefore do not establish a direct effect of intestinal GLP-1R activation on pathogen elimination. Live-pathogen models and tissue-specific genetic deletion are needed to determine the receptor-dependent contribution of this pathway to infectious enteritis, antibiotic-associated dysbiosis, and gut-derived sepsis.

In the lung, GLP-1R expression or associated signalling has been reported in alveolar and airway epithelial cells, airway smooth muscle, and pulmonary vascular compartments [32]. This distribution provides a putative anatomical basis for GLP-1RAs to influence alveolar epithelial function, surfactant production, airway inflammation, pulmonary vascular responses, and alveolar–capillary barrier integrity. Nevertheless, pulmonary protection during infection or sepsis may not be mediated exclusively by local lung GLP-1R. Animal studies indicate that central neuronal GLP-1R activation can limit systemic inflammation and reduce lung injury in polymicrobial sepsis [9]. Pulmonary protection may therefore reflect the combined effects of local epithelial or vascular signalling, central neuroimmune regulation, and systemic metabolic improvement. Reduced inflammatory markers alone do not establish lung-cell-intrinsic GLP-1R signalling.

In cardiovascular and renal tissues, GLP-1R expression is generally low and highly dependent on species, anatomical compartment, and detection methodology [26, 27, 33, 34]. In cardiovascular disease and chronic kidney disease, GLP-1RAs may confer protection by improving endothelial function, reducing oxidative stress and inflammation, and preserving microcirculatory homeostasis [35–37]. Because myocardial and renal injury in severe infection is driven partly by endothelial dysfunction, impaired microvascular perfusion, mitochondrial stress, and systemic inflammation, these cardiorenal mechanisms may improve organ tolerance and limit secondary injury [38, 39]. These effects, however, are extrapolated mainly from non-infectious cardiorenal disease and should therefore be regarded as putative mechanisms of host tolerance and organ protection.

The central nervous system may represent a key integrative hub through which GLP-1R activation regulates systemic inflammation [5, 9, 40]. GLP-1R expression in the hypothalamus, nucleus tractus solitarius, dorsal motor nucleus of the vagus, and other autonomic regulatory regions enables these neural circuits to integrate metabolic, stress-related, and inflammatory signals and thereby coordinate responses in peripheral organs [5, 27]. Cell-specific genetic studies show that GLP-1R agonism attenuates circulating TNF responses to multiple Toll-like receptor agonists and improves sickness behaviour, hypothermia, systemic inflammation, and lung injury during polymicrobial sepsis [9]. These protective effects are substantially diminished by neuronal *Glp1r* deletion but remain intact after *Glp1r* deletion in haematopoietic or endothelial cells. This genetic pattern supports a central neuronal GLP-1R contribution to the regulation of peripheral inflammation and infection-associated organ injury through autonomic and neuroimmune pathways [9].

Outside the central nervous system, GLP-1R agonism may influence infection-related outcomes through several direct and indirect pathways. Pancreatic β-cell GLP-1R activation may improve the systemic metabolic milieu by ameliorating hyperglycaemia, insulin resistance, and lipid dysfunction. GLP-1R-associated signalling in gastrointestinal and pulmonary tissues may contribute to epithelial and barrier homeostasis, whereas cardiorenal actions may support microcirculatory function and organ tolerance [5, 8]. These peripheral actions may attenuate metabolic stress, inflammatory amplification, and barrier failure during infection, but their dependence on local tissue GLP-1R requires validation through cell-resolved, disease-specific, and genetic approaches [31, 36, 41, 42].

In contrast to the well-established receptor biology in pancreatic β cells and defined neuronal populations, the presence and biological relevance of functional GLP-1R in immune cells remain poorly defined. Although interventions targeting the GLP-1/GLP-1R axis have been associated with changes in macrophage polarisation, inflammatory phenotypes, and cytokine expression, most evidence derives from metabolic or sterile inflammatory models and does not establish macrophage-intrinsic receptor signalling [8, 43]. Given the central and systemic actions described above, treatment-associated macrophage phenotypes may instead reflect neuroimmune regulation, metabolic improvement, endothelial modulation, or remodelling of the local tissue microenvironment [8, 9]. Accordingly, such phenotypes should not be attributed to cell-intrinsic GLP-1R activation without myeloid-specific *Glp1r* manipulation and direct demonstration of receptor-dependent signalling.

Claims regarding GLP-1R expression and function in adaptive immune cells also warrant particular caution. A 2024 study identified GLP-1R-associated signals in selected T-cell subsets and reported enrichment of exhaustion-like CD8⁺ T cells within antibody-sorted GLP-1R-positive populations [44]. On this basis, the authors proposed that GLP-1R may function as a negative co-stimulatory regulator of T-cell activation and alloimmunity [44]. Receptor expression was assessed using multiple approaches, including flow cytometry, Western blotting, RT-qPCR, Sanger sequencing of PCR products, bulk transcriptomics, and single-cell RNA sequencing, rather than being inferred from transcriptomic data alone [44]. However, both protein detection and isolation of GLP-1R-positive T cells depended largely on the AGR-021 antibody. A 2025 reassessment used two independent GLP-1R knockout mouse lines to test AGR-021 specificity [10]**.** AGR-021 retained immunoreactivity in receptor-deficient islets and splenic T cells and produced non-specific Western blot bands, challenging its suitability for identifying or sorting GLP-1R-positive T cells [10]. In this context, RT-qPCR and Sanger sequencing support only the presence of GLP-1R transcripts in some T cells and do not establish a cell-surface receptor with specific ligand-binding and signalling competence [10, 33, 45]. The proposed enrichment of GLP-1R in exhaustion-like CD8⁺ T cells, the existence of an intracellular receptor reservoir, and receptor-mediated negative co-stimulation therefore remain provisional rather than established features of T-cell biology [10, 44]. Definitive validation will require knockout-verified antibodies and specific fluorescent or radioligand-binding approaches [10, 33, 46]. Endogenous reporter or knock-in systems, receptor-dependent second-messenger assays, and T-cell-specific GLP-1R loss- or gain-of-function approaches should provide complementary functional and genetic evidence [34, 47]. Pending such validation, treatment-associated changes in T-cell activation, exhaustion, cytokine production, or effector activity do not by themselves establish T-cell-intrinsic GLP-1R signalling. Existing T-cell evidence also comes largely from transplantation, cancer immunity, and sterile inflammatory contexts, leaving its relevance to acute viral infection, chronic infection, and sepsis-associated immune suppression unresolved [8, 44].

Evidence for functional, receptor-dependent GLP-1R signalling in neutrophils and dendritic cells is even less developed. Available data do not establish that GLP-1RAs directly enhance neutrophil chemotaxis, phagocytosis, oxidative burst, or microbial killing through a cell-intrinsic receptor, nor that they regulate dendritic-cell antigen presentation, co-stimulation, or T-cell priming through a receptor-autonomous mechanism [8]. Neutrophil and dendritic-cell phenotypes observed after GLP-1RA treatment may also reflect indirect effects of improved metabolic control, central neuroimmune signalling, reduced systemic inflammation, and tissue-microenvironment remodelling [8, 9].

GLP-1R localisation studies are further constrained by major methodological limitations. Low receptor abundance, strong cell-type and regional specificity, and inadequate knockout validation of some commercial antibodies make immunohistochemistry, immunofluorescence, or Western blotting alone vulnerable to non-specific detection and erroneous cellular assignment [10, 33, 34]. Single-cell RNA sequencing may under-detect low-abundance transcripts because of inefficient capture and dropout, meaning that absence of a *GLP-1R* signal does not establish true receptor absence [48]. Conversely, transcript detection confirms only that *GLP-1R* RNA is present and does not prove surface expression of a functional receptor, particularly because transcript and protein levels may be discordant [45].

Reliable assignment of immune-cell GLP-1R therefore requires convergent evidence from RNA in situ hybridisation, knockout-validated antibodies, and specific ligand-binding approaches [10, 33, 46]. Endogenous reporter or knock-in systems, receptor-dependent signalling assays, and cell-specific GLP-1R deletion or rescue should provide complementary functional and genetic validation [34, 47]. In infection models, high-purity cell isolation should be used in live-pathogen experiments together with parallel assessment of microbial burden, phagocytosis and killing, antigen presentation, cytokine responses, and tissue injury.

GLP-1R function in infection is better conceptualised as an integrated central–peripheral network than as a discrete anti-inflammatory pathway within individual immune cells. Within this network, neuronal GLP-1R may restrain systemic inflammation through neuroimmune reflexes, while β cells and metabolic tissues may support host defence indirectly by improving the metabolic milieu [8, 9]. Peripheral organs may preserve barrier integrity and organ tolerance, whereas direct receptor effects in selected immune-cell subsets remain possible but unconfirmed [8, 9]. These axes are likely to interact within a coordinated metabolic–neural–immune network. Resolving the relative contributions of central neuroimmune regulation, peripheral tissue protection, systemic metabolic improvement, and direct immune-cell signalling will require cell-specific genetic deletion, neural-circuit tracing, ligand-binding studies, single-cell and spatial omics, and live-pathogen models across pathogen types, infection stages, and host metabolic phenotypes (Table 1).

**Table 1:** Evidence hierarchy for GLP-1R assignment and GLP-1R-associated effects across tissues and immune cells

Tissue/cell	Evidence grade and key evidence	Infection relevance and limitation	References
Pancreatic β cells	Established. Consistent transcript, ligand-binding and receptor-dependent cAMP/insulin responses	Improves glycaemia and systemic metabolic stress; supports host defence indirectly rather than through an immune-cell mechanism	[5, 27]
Central neurons	Established. Neuronal Glp1r loss attenuates GLP-1RA effects on TNF, hypothermia and lung injury in polymicrobial sepsis	Supports neuroimmune regulation of systemic inflammation and organ injury; evidence is predominantly preclinical	[9]
Intestinal compartments	Context-dependent. Receptor-associated effects on motility, colonic metabolism, goblet cells and epithelial integrity	Possible mucus and barrier support; most evidence derives from metabolic or sterile-injury models	[49]
Pulmonary compartments	Suggestive. Pharmacological effects on alveolar injury, surfactant proteins and epithelial-barrier phenotypes	Possible lung-barrier and surfactant protection; the local receptor contribution remains unresolved	[13, 32]
Endothelium	Context-dependent. Cell-specific Glp1r deletion supports vascular receptor function in selected non-infectious settings	May influence permeability, vascular tone and microcirculation; effects vary by vascular bed and disease context	[50]
Macrophages	Suggestive. Polarisation, migration and cytokine phenotypes are reported mainly in cell, metabolic or sterile-inflammation models	Potential inflammation/repair effects, but macrophage-intrinsic receptor action in infection remains unproven	[51, 52]
T cells	Disputed. Candidate negative co-stimulation was reported, but AGR-021-based receptor detection was challenged in Glp1r-null tissues	Potential effects on activation or immunopathology remain provisional and require receptor-specific validation	[10, 44]
Neutrophils and dendritic cells	Insufficient. No reproducible receptor-dependent evidence for microbial killing, antigen presentation or T-cell priming	Observed phenotypes should be regarded as potentially indirect until purified-cell and cell-specific genetic evidence is available	[8]

Evidence grades reflect the convergence of transcript localisation, knockout-validated protein detection, specific ligand binding, receptor-dependent signalling, and cell-specific genetic evidence. Established indicates convergent functional or genetic evidence; context-dependent indicates reproducible evidence that varies by tissue, species, or method; suggestive indicates predominantly pharmacological or indirect evidence; disputed indicates conflicting receptor assignment; and insufficient indicates no convincing receptor-dependent evidence

### GLP-1R signalling

Following consideration of GLP-1R distribution and the associated methodological challenges, attention should turn to its canonical signalling programme and its extensive crosstalk with metabolic homeostasis, immune regulation, and barrier protection. The core molecular architecture of canonical GLP-1R signalling has been well characterised [5]. Upon ligand binding, GLP-1R undergoes a conformational change and couples to Gαs, which activates adenylyl cyclase and rapidly increases intracellular cAMP. cAMP signals predominantly through PKA and Epac [5, 28, 53]. In pancreatic β cells, PKA-dependent phosphorylation of transcription factors including CREB promotes insulin gene expression and glucose-dependent secretion, whereas Epac activates small GTPases such as Rap1 to facilitate insulin-granule recruitment and exocytosis [28, 53]. The Gαs–cAMP–PKA/Epac pathway, with CREB as a major PKA-responsive transcriptional effector, forms the canonical molecular basis of GLP-1R-mediated metabolic actions [5].

Beyond cAMP-dependent signalling, GLP-1R engages in extensive crosstalk with PI3K/AKT, MAPK/ERK, AMPK, and mTOR networks, with PI3K/AKT and MAPK/ERK signalling potentially supporting cell survival, resistance to apoptosis, stress adaptation, and tissue repair during infection-associated injury [5]. GLP-1R activation may also alter intracellular Ca2⁺ dynamics and AMPK phosphorylation, thereby linking cellular energy sensing and metabolic remodelling to inflammatory regulation [5, 54].

**Fig. 1:**
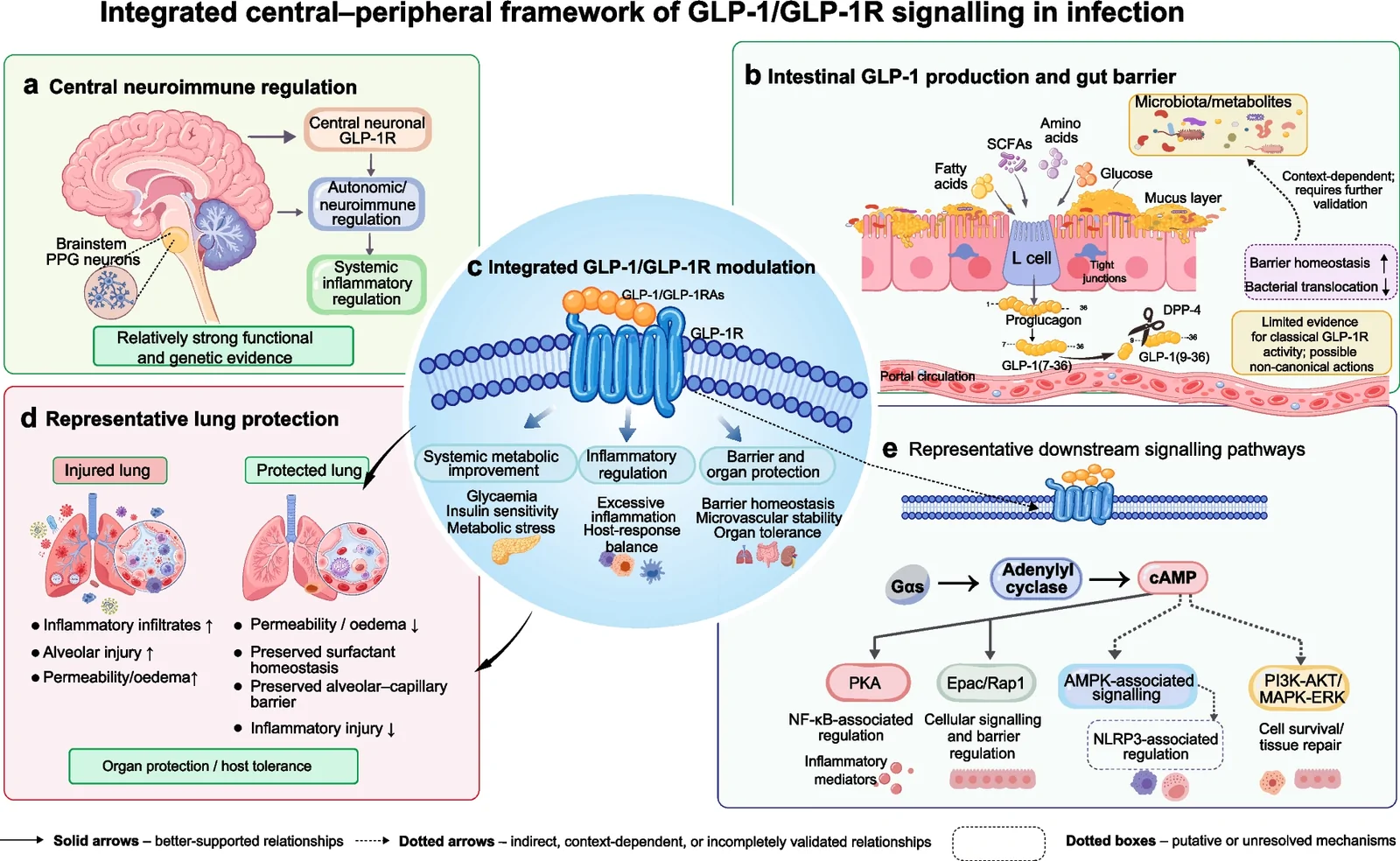
Integrated central–peripheral framework of GLP-1/GLP-1R signalling in infection. **a** Central neuroimmune regulation through brainstem PPG neurons, neuronal GLP-1R signalling, and autonomic pathways. **b** Intestinal GLP-1 production and gut-barrier regulation involving nutrient- and microbiota-derived signals, proglucagon processing, DPP-4-mediated degradation, and barrier homeostasis. **c** Integrated GLP-1/GLP-1R modulation of systemic metabolism, inflammation, barrier function, and organ protection. **d** The lung illustrates potential organ-protective effects on inflammatory injury, surfactant homeostasis, and preservation of the alveolar–capillary barrier. **e** Representative downstream pathways include Gαs–adenylyl cyclase–cAMP signalling through PKA and Epac/Rap1, with context-dependent crosstalk involving AMPK, PI3K–AKT, MAPK–ERK, NF-κB, and NLRP3. Solid arrows indicate better-supported relationships; dotted arrows indicate indirect, context-dependent, or incompletely validated relationships; dotted boxes denote putative or unresolved mechanisms. The figure was redrawn by the authors, with graphical elements adapted from BioGDP. Abbreviations: AKT, protein kinase B; AMPK, AMP-activated protein kinase; cAMP, cyclic adenosine monophosphate; DPP-4, dipeptidyl peptidase-4; Epac, exchange protein directly activated by cAMP; ERK, extracellular signal-regulated kinase; GLP-1, glucagon-like peptide-1; GLP-1R, glucagon-like peptide-1 receptor; GLP-1RA, GLP-1 receptor agonist; Gαs, stimulatory G protein alpha subunit; MAPK, mitogen-activated protein kinase; NF-κB, nuclear factor κB; NLRP3, NLR family pyrin domain containing 3; PI3K, phosphoinositide 3-kinase; PKA, protein kinase A; PPG, preproglucagon; Rap1, Ras-related protein 1; SCFAs, short-chain fatty acids

These signalling interactions may be particularly relevant to the pathophysiology of infection. The cAMP–PKA pathway may intersect with NF-κB-dependent transcription to modulate pro-inflammatory mediators such as TNF-α and IL-6 [55]. AMPK activation may further limit sepsis-associated tissue injury by restraining NLRP3 inflammasome activation [56, 57]. In parallel, PI3K/AKT and MAPK signalling may support infection-associated organ protection by preserving epithelial and endothelial barrier integrity and enhancing cell survival [5]. The cAMP–PKA/Epac core pathway and its interactions with AMPK, PI3K/AKT, MAPK, NF-κB, and NLRP3 may allow GLP-1R activation to coordinate metabolic sensing, inflammatory control, barrier preservation, and organ tolerance within an integrated immunometabolic network. Thus, canonical GLP-1R signalling is more appropriately conceptualised not simply as a glucose-lowering pathway but as a multidimensional molecular network connecting metabolic homeostasis with immune regulation, barrier integrity, and organ tolerance (Fig. 1).

## Mechanisms linking GLP-1R signalling to host defence

Having established the sources of GLP-1, the tissue distribution of GLP-1R, and its canonical signalling pathways, this section examines how the GLP-1/GLP-1R axis may mechanistically shape host defence during infection [5]. These potential effects are discussed across immunometabolic regulation, innate and adaptive immune responses, inflammatory signalling, barrier protection, and autophagy–mitochondrial homeostasis, with central neuroimmune regulation considered as an important cross-cutting mechanism [9, 36, 49]. Given the lack of consistent validation of functional GLP-1R expression in immune cells, immune phenotypes following GLP-1RA treatment should be interpreted in systemic metabolic, neuroimmune, and local tissue contexts; by themselves, these phenotypes do not establish immune-cell-intrinsic GLP-1R activation [8, 10].

### Immunometabolic regulation

Immunometabolic regulation describes the dynamic reconfiguration of glycolysis, oxidative phosphorylation (OXPHOS), fatty acid oxidation (FAO), amino acid metabolism, and mitochondrial bioenergetics in immune cells [58]. During infection, inflammation, hypoxia, and tissue injury, these programmes are reconfigured to meet the energetic and biosynthetic requirements of activation, effector activity, inflammation resolution, and tissue repair [58]. The functional consequences of these metabolic programmes depend on immune-cell identity, infection stage, and the surrounding tissue microenvironment [58, 59]. The same pathway may therefore exert distinct or even opposing effects across cellular and disease contexts; glycolysis, OXPHOS, and FAO should not be assigned fixed pro-inflammatory or anti-inflammatory roles in isolation [58, 59]. At the molecular level, AMP-activated protein kinase (AMPK), mammalian target of rapamycin (mTOR), and hypoxia-inducible factor-1α (HIF-1α) function as major sensors of cellular energy availability, nutrient and growth-factor signalling, and oxygen tension, respectively, thereby integrating metabolic adaptation with immune-cell functional states [60].

Hyperglycaemia, insulin resistance, and obesity may create an unfavourable metabolic and immunological state before pathogen exposure occurs. Sustained hyperglycaemia impairs neutrophil chemotaxis, phagocytosis, oxidative burst, and microbial killing and may additionally compromise monocyte–macrophage-mediated pathogen control, T-cell function, and vascular endothelial homeostasis [61, 62]. Insulin resistance and obesity are further characterised by lipotoxicity, chronic low-grade inflammation, oxidative stress, and vascular dysfunction, collectively creating conditions that may impair host resistance and increase both susceptibility to infection and the risk of severe disease [63, 64]. Accordingly, the adverse effects of diabetes and obesity on infection outcomes do not arise from an isolated immune-cell defect but reflect interacting abnormalities in immunometabolic regulation, tissue-barrier integrity, and microcirculatory function [61, 65].

Through enhanced glucose-dependent insulin secretion, suppression of inappropriate glucagon release, delayed gastric emptying, and reduced energy intake, GLP-1R activation improves glycaemic control, insulin sensitivity, body weight, and selected indices of lipid metabolism [5, 66]. In infection, the most strongly supported interpretation is that these established metabolic actions indirectly create a more favourable systemic environment for immune cells, endothelial cells, and epithelial barrier cells by reducing exposure to hyperglycaemia, insulin resistance, lipotoxicity, and chronic metabolic inflammation [5, 8]. Immune and inflammatory phenotypes observed after GLP-1RA treatment therefore do not, by themselves, establish immune-cell-intrinsic metabolic reprogramming. Systemic changes in glycaemia, body weight, food intake, adipose-tissue inflammation, and neuroendocrine regulation may also contribute to these phenotypes.

Studies in metabolic disease and sterile inflammatory models suggest that GLP-1/GLP-1R signalling may interact with cellular networks that regulate energy sensing, nutrient responsiveness, and mitochondrial homeostasis, thereby shaping cellular adaptation to stress and subsequent functional states [5, 8]. However, it remains unclear whether these mechanisms operate directly within immune cells under infectious conditions, and extrapolation from non-infectious models therefore requires caution. Changes in metabolic signalling proteins, inflammatory mediators, or static metabolic markers do not by themselves establish immune-cell metabolic reprogramming. Stronger evidence requires functional measurements of glycolysis and mitochondrial respiration, together with analyses of substrate utilisation, metabolic flux, or metabolomic profiles. Cell-specific receptor manipulation with glycaemia-, body-weight-, and food-intake-matched controls is also needed to distinguish cell-intrinsic effects from systemic influences.

Interpretation of infection outcomes must also distinguish between enhanced host resistance, defined by improved pathogen control or clearance, and enhanced host tolerance, in which tissue injury is limited and organ function preserved despite little or no reduction in pathogen burden [67, 68]. The available evidence more strongly supports indirect modulation of infection-associated immunometabolism by GLP-1RAs through improvement of the systemic metabolic milieu [5, 8, 9]. Whether immune-cell-intrinsic GLP-1R directly drives metabolic reprogramming remains unresolved. Addressing this question, and determining whether any such effect primarily alters host resistance or host tolerance, will require infection models that combine functional metabolic profiling with cell-specific receptor validation [5, 8, 9].

### Innate immunity

Innate immune cells coordinate early pathogen control and subsequent tissue repair through pathogen recognition, chemotactic recruitment, phagocytosis and microbial killing, regulation of inflammation, and antigen presentation [69]. Available evidence suggests that GLP-1/GLP-1R-related interventions may alter the recruitment, tissue localisation, and functional states of macrophages, neutrophils, and dendritic cells, but the evidential support is uneven across these populations [5, 70]. Macrophage-related evidence is comparatively abundant, whereas direct receptor-dependent effects in neutrophils and dendritic cells remain poorly established [8]. Given that the methodological challenges surrounding immune-cell GLP-1R expression have been addressed in the section on GLP-1R distribution and functional validation, the present section focuses on innate immune phenotypes observed after GLP-1RA treatment and their potential significance during infection.

Among innate immune populations, macrophages have received the greatest attention in studies of GLP-1-related immune regulation [36]. In RAW264.7 cells, GLP-1 treatment has been associated with reduced inflammatory phenotypes and increased expression of selected markers linked to tissue repair [51]. In monosodium urate-induced sterile peritonitis, genetic and pharmacological manipulation of GLP-1R-related signalling altered macrophage trafficking to inflamed tissue, implicating this axis in cellular recruitment and localisation [52]. In metabolic inflammatory settings such as atherosclerosis, GLP-1RA treatment has also been linked to reduced monocyte–macrophage infiltration, lower intracellular lipid accumulation, and restricted foam-cell formation [71]. Collectively, these observations identify macrophages as important functional responders to GLP-1RA treatment, with potential changes spanning recruitment, migration, lipid handling, and the balance between inflammatory and reparative states [72]. However, because these findings arise predominantly from cell lines and metabolic or sterile-inflammatory models, they do not establish direct protective reprogramming of macrophages through cell-intrinsic GLP-1R signalling in infectious settings [8].

Macrophage function is highly plastic and is shaped by the surrounding tissue environment, favouring a broad spectrum of activation states rather than a binary and mutually exclusive “M1/M2” classification [73]. Consequently, lower inflammatory markers or higher repair-associated markers after GLP-1RA treatment signify a shift in macrophage state but do not establish that phagocytosis, intracellular killing, or pathogen clearance has improved in parallel [74]. The biological consequences of such changes are also likely to vary according to the anatomical site and temporal stage of infection. Macrophage recruitment and microbicidal activity may support pathogen containment early in infection, whereas restraint of sustained activation during inflammatory amplification or tissue repair may instead limit immunopathological injury [75].

Compared with macrophages, neutrophils have received substantially less attention in studies of GLP-1-related intervention, although some studies provide direct phenotypic and functional observations. Clinical and in vitro evidence indicates that semaglutide treatment may alter neutrophil phenotypes and modify CD11b expression, chemotaxis, and endothelial adhesion in neutrophil-like cells [76]. The reported changes are consistent with altered neutrophil trafficking from the circulation into inflamed tissues rather than a simple change in circulating cell numbers. Most of this evidence, however, comes from atherosclerosis and metabolic inflammation, and live-pathogen studies have not systematically assessed neutrophil chemotaxis, phagocytosis, oxidative burst, neutrophil extracellular trap formation, or microbial killing [70]. Whether such changes enhance or impair early pathogen control remains unresolved.

Evidence linking GLP-1-related intervention to dendritic cells is centred largely on their accumulation in inflamed tissues and on phenotypes related to antigen presentation. In experimental autoimmune encephalomyelitis, dulaglutide reduced the accumulation of dendritic cells and macrophages in the central nervous system, raising the possibility that GLP-1RAs modify the recruitment or persistence of antigen-presenting cells in inflamed tissues [77]. In a type 1 diabetes immunotherapy setting, liraglutide preserved the phenotype and antigen-presentation-related functions of tolerogenic dendritic cells, indicating that GLP-1RA treatment need not interfere with dendritic-cell-mediated immune regulation [78]. Neither study directly examined whether GLP-1RAs alter dendritic-cell uptake, processing, or presentation of microbial antigens, nor did they define effects on co-stimulation and downstream immune priming during infection [70]. These observations should be interpreted as mechanistic clues that GLP-1RAs may modify dendritic-cell trafficking and functional state, rather than as evidence of direct anti-infective efficacy.

Across innate immune populations, GLP-1-related phenotypes encompass macrophage recruitment, migration, lipid handling, and inflammatory–reparative states; neutrophil CD11b expression, chemotaxis, and endothelial adhesion; and dendritic-cell accumulation and antigen-presentation-associated features within inflamed tissues [36]. A critical distinction must be made between cells that exhibit secondary functional changes after treatment and the primary cellular targets of GLP-1RAs, because many of these phenotypes may result indirectly from improved systemic metabolism or a less inflammatory tissue milieu [79]. The extent to which these effects require cell-intrinsic GLP-1R signalling in macrophages, neutrophils, or dendritic cells remains unresolved [9]. Future work should integrate cell-type-specific Glp1r manipulation and matched functional assays in live-pathogen models to define where GLP-1-related regulation acts within the innate immune response and whether it ultimately alters pathogen control, tissue injury, or both [8].

### Adaptive immunity

Adaptive immunity is essential for pathogen clearance, the generation of immunological memory, and the regulation of infection-associated tissue injury; however, the functions of individual CD4⁺ and CD8⁺ T-cell subsets vary substantially across acute infection, chronic infection, and the recovery phase [80]. T-cell activation, clonal expansion, effector differentiation, and memory formation require coordinated changes in glycolysis, mitochondrial oxidative metabolism, fatty acid utilisation, and biosynthetic pathways [80, 81]. Any influence of GLP-1/GLP-1R signalling on adaptive immunity must therefore be evaluated in the context of T-cell lineage, activation or exhaustion state, infection stage, and the host metabolic milieu [80, 81].

Indirectly, hyperglycaemia, insulin resistance, and the chronic inflammatory state associated with obesity may disrupt T-cell bioenergetics, redox balance, and mitochondrial function, thereby compromising proliferation, effector activity, and immune homeostasis [82, 83]. By improving glycaemic control, insulin sensitivity, body weight, and systemic inflammatory tone, GLP-1RAs may create a metabolic environment that is more permissive for T-cell activation, effector function, and memory formation. This interpretation is supported mainly by the established systemic metabolic effects of GLP-1RAs and by the sensitivity of T-cell function to the surrounding metabolic environment [5, 80, 82]. Current evidence does not establish that GLP-1RAs directly enhance pathogen-specific T-cell responses during infection. In obese diabetic db/db mice, exenatide was associated with lower Th17-cell frequencies in peripheral blood and the spleen and with an increased proportion of regulatory T cells, changes that were proposed to involve PI3K/Akt/FoxO1 signalling [84]. These findings were obtained in a non-infectious metabolic disease model. They show an association between exenatide treatment and altered Th17/Treg balance but do not establish the contribution of T-cell-intrinsic GLP-1R or predict whether this shift would improve pathogen clearance during infection.

With respect to potential direct effects, evidence from transplantation and alloimmune settings has raised the possibility that GLP-1R may act as a negative co-stimulatory receptor in T cells. Antibody-based sorting identified enrichment of exhaustion-like CD8⁺ T cells within the putative GLP-1R-positive population, and GLP-1R-related intervention was associated with weaker alloreactive T-cell responses and reduced intragraft T-cell infiltration [44]. These observations raise the possibility that, in T-cell subsets expressing signalling-competent receptor, direct GLP-1R activation may constrain excessive effector activity during persistent or dysregulated immune stimulation and consequently limit immunopathological injury [8]. However, because the evidence derives from transplantation and alloimmune responses, it should presently be viewed as a candidate mechanism for direct T-cell GLP-1R activity rather than an established principle of infection biology. Its relevance to acute viral infection, persistent infection, and sepsis-associated immunosuppression remains to be established [10, 44].

At the molecular level, GLP-1R couples to Gαs, and functional expression in selected T-cell subsets would be expected to increase intracellular cAMP and potentially modify T-cell receptor signalling, activation thresholds, cytokine production, and differentiation through PKA-, Epac-, and CREB-associated pathways [85–87]. Although cAMP-dependent pathways can regulate TCR signalling, activation thresholds, cytokine production, and differentiation, direct mediation of these effects by T-cell GLP-1R has not yet been demonstrated. The functional consequences of cAMP signalling in T cells further depend on signal amplitude, subcellular compartmentalisation, the available effector machinery, and the cellular activation state. Although cAMP–PKA signalling is commonly linked to inhibition of TCR-driven activation, spatially distinct signalling microdomains and varying contributions from PKA, Epac, and other effectors may generate context-dependent or even divergent responses [85, 86]. Thus, Gαs coupling offers a plausible signalling framework but does not justify assuming that GLP-1R uniformly suppresses or enhances T-cell function.

Overall, the evidence favours a bidirectional and context-dependent model rather than a uniform effect on adaptive immunity. By improving hyperglycaemia, insulin resistance, obesity-associated inflammation, and mitochondrial metabolism, GLP-1RAs may indirectly support T-cell metabolic fitness. In parallel, direct GLP-1R signalling in selected T-cell populations could restrain excessive activation and immunopathology, but its receptor dependence and T-cell-subset specificity remain insufficiently resolved [10, 44, 80]. The balance between these indirect and direct effects is likely to vary with T-cell subset, pathogen and disease stage, host metabolic state, and treatment timing, precluding a simple classification of GLP-1/GLP-1R signalling as uniformly stimulatory or inhibitory to adaptive immunity [10, 44]. Future work should integrate T-cell-specific Glp1r deletion and rescue, receptor-dependent cAMP assays, TCR clonotype tracking, single-cell and spatial omics, metabolic-flux analysis, and live-pathogen models, with parallel assessment of pathogen burden, effector function, immunopathology, and memory development.

### Inflammatory signalling and inflammasomes

Inflammation exerts context-dependent and potentially opposing effects during infection, as a proportionate response promotes effector-cell recruitment, restricts pathogen spread, and initiates tissue repair, whereas persistent or dysregulated inflammation drives endothelial injury, vascular leakage, coagulation abnormalities, and multiple-organ dysfunction [88, 89]. TNF-α, IL-6, and IL-1β participate in endothelial activation, leucocyte recruitment, and acute-phase responses, but their effects vary with signal magnitude, duration, and infection stage, while excessive counter-regulation or immunosuppression may conversely compromise protective immunity and pathogen clearance [88]. On this basis, the anti-inflammatory effects associated with GLP-1/GLP-1R signalling should be evaluated against two complementary criteria: attenuation of inflammatory tissue injury and preservation of effective anti-infective immunity.

At the transcriptional level, this inflammatory balance is shaped by NF-κB, MAPK, and JAK/STAT signalling pathways [89]. NF-κB and MAPK contribute to inflammatory gene induction and signal amplification [90], whereas STAT pathways integrate signals from IL-6-family cytokines, interferons, and other mediators to regulate acute-phase responses, antimicrobial gene programmes, and immune feedback mechanisms [91, 92]**.** These signalling pathways are extensively interconnected, and the magnitude, duration, and transcriptional consequences of their activation are shaped collectively by ligand identity, cellular context, pathogen properties, and infection stage [89, 93]. As a Gαs-coupled receptor, GLP-1R can engage cAMP–PKA/Epac signalling, which may intersect with AMPK, PI3K/AKT, and MAPK networks [5, 8]. These interactions may modify inflammatory transcriptional programmes, but their effects depend on functional receptor expression in the target cell and on the surrounding metabolic and inflammatory context [5, 8]. Cytokine and chemokine changes observed after GLP-1RA treatment may therefore represent the composite effects of local receptor signalling, systemic metabolic improvement, and tissue-microenvironment remodelling rather than a single cell-autonomous mechanism [36].

The NLRP3 inflammasome provides a critical link between inflammatory priming and the subsequent maturation and release of IL-1β and IL-18. Canonical NLRP3 inflammasome activation is generally described as a two-step process [94, 95]. During the priming step, Toll-like receptor or cytokine-receptor signalling activates NF-κB and increases the expression of NLRP3, pro-IL-1β, and related components. A second activation step is triggered by cellular stress signals, including K⁺ efflux, lysosomal injury, disruption of Ca2⁺ homeostasis, reactive oxygen species accumulation, and mitochondrial dysfunction. These signals promote inflammasome assembly, caspase-1 activation, and the release of mature IL-1β and IL-18 [94, 95]. Because the relative contribution of these signals varies across cell types and infection models, NLRP3 or pro-IL-1β expression alone cannot establish functional inflammasome activation; assessment should additionally include ASC aggregation, caspase-1 activation, and the release of mature cytokines [95].

Interventions targeting the GLP-1/GLP-1R axis may indirectly modulate NLRP3 inflammasome activity by modifying NF-κB-dependent priming, cellular redox balance, and mitochondrial stress. NRF2 is a major transcriptional regulator of cellular responses to oxidative and electrophilic stress, inducing HO-1, NQO1, GCLC, and other genes involved in antioxidant defence and glutathione metabolism to enhance cellular resilience to oxidative stress [96]. Evidence from metabolic and sterile injury models indicates that GLP-1-related interventions may enhance NRF2-associated antioxidant responses, with some studies concurrently reporting reductions in NF-κB, NLRP3, caspase-1, IL-1β, and IL-18-related readouts [97, 98]. These observations establish an association between GLP-1-related tissue protection, changes in oxidative stress, and altered NLRP3 activity [99, 100]. However, they do not yet demonstrate, in live-pathogen infection models, a complete causal sequence from GLP-1R activation through NRF2 enhancement and NLRP3 suppression to improved infection outcomes [97, 98].

A clear distinction between direct and indirect mechanisms is fundamental to the interpretation of GLP-1RA-associated anti-inflammatory phenotypes. Reduced tissue readouts related to NF-κB or NLRP3 may reflect local receptor signalling, but they may equally arise downstream of improved metabolic status, reduced tissue injury, or central neuroimmune regulation [36, 99]. Cell-specific genetic evidence indicates that neuronal Glp1r deletion diminishes several systemic anti-inflammatory effects of GLP-1R agonism, whereas comparable deletion in haematopoietic or endothelial cells does not, implicating central neuroimmune pathways in the regulation of peripheral inflammatory networks [9]. Decreases in peripheral cytokines, NF-κB-associated readouts, or inflammasome-related markers after GLP-1RA treatment do not, by themselves, establish direct immune-cell-intrinsic GLP-1R signalling.

GLP-1/GLP-1R signalling may shape cytokine, chemokine, and NLRP3-associated responses through coordinated effects on inflammatory transcriptional networks, the metabolic and redox environment, and central neuroimmune communication [36]. Current data more strongly support systemic anti-inflammatory and tissue-protective effects, whereas direct regulation of NRF2 or NLRP3 by immune-cell-intrinsic GLP-1R remains plausible but unconfirmed [8, 98]. Determining whether reduced cytokine or inflammasome activity reflects improved host resistance, attenuation of immunopathological injury, or non-specific suppression of inflammation requires parallel assessment of pathogen burden, clearance, immune effector competence, and organ injury [100]. Live-pathogen models with post-infection treatment and cell-type-specific Glp1r manipulation would allow these mechanisms to be evaluated together with inflammasome assembly, caspase-1 activity, mature IL-1β and IL-18 release, pathogen control, and organ function [101].

### Barrier protection

Infection-associated barrier disruption is not only a consequence of local inflammation, exposure to microbial products, oxidative stress, and cellular injury but also a major contributor to microbial translocation, tissue oedema, and the amplification of systemic inflammation [102, 103]. The intestinal epithelium, alveolar–capillary interface, and vascular endothelium form interconnected structural barriers that limit pathogen dissemination and preserve organ homeostasis, relying respectively on epithelial tight junctions and mucus, the pulmonary surfactant system, and the endothelial glycocalyx and microcirculatory control [104–107].

In the intestinal barrier, claudins, occludin, and zonula occludens-1 (ZO-1) regulate paracellular permeability, while the goblet-cell-derived MUC2 mucus layer and continuous epithelial renewal jointly preserve mucosal integrity [104, 108]. GLP-1/GLP-1R signalling may support intestinal barrier homeostasis by modulating epithelial metabolic adaptation, cell survival, tight-junction stability, and mucus-barrier maintenance, with the PI3K/AKT/HIF-1α axis representing a candidate mechanistic link between GLP-1 signalling and epithelial integrity [49, 109]. However, most available findings derive from metabolic or sterile injury models, and whether GLP-1-related barrier effects limit the translocation of pathogens or microbial products during infection remains to be established using live-pathogen models and functional measures of barrier integrity [109]**.**

GLP-1 signalling is also embedded within a bidirectional network involving the gut microbiota, short-chain fatty acids, bile acid metabolism, and gastrointestinal motility. Microbiota-derived metabolites can regulate GLP-1 secretion by intestinal L cells, while GLP-1RAs may reciprocally reshape microbial composition and metabolite profiles through changes in gastric emptying, nutrient delivery, and the intestinal milieu [18, 110]. Because current studies predominantly report parallel changes in microbial composition, metabolite profiles, and barrier phenotypes, microbiota remodelling should presently be considered a potential mediator of GLP-1-related barrier regulation; its causal contribution to protection during infection remains unresolved [110]**.** Interpretation of intestinal barrier studies requires a clear hierarchy of evidence. mRNA changes indicate transcriptional regulation, whereas total-tissue protein measurements indicate abundance [111]. Junctional-protein localisation, permeability, and microbial translocation provide more direct evidence of functional barrier integrity. Integrating structural readouts with functional outcomes therefore provides a stronger basis for concluding that barrier function has been restored [111].

Within the alveolar–capillary barrier, tight and adherens junctions between alveolar epithelial and pulmonary capillary endothelial cells restrict the movement of fluid and plasma proteins into the alveolar airspace [112]. Alveolar type II epithelial cells are central to barrier maintenance, serving both as the principal source of pulmonary surfactant and as progenitor cells that proliferate and contribute to epithelial repair after alveolar injury [113, 114]. Within the pulmonary surfactant system, SP-B promotes the formation and stability of surfactant films and thereby reduces alveolar surface tension, whereas SP-A contributes to surfactant homeostasis, pathogen recognition, and innate immune regulation within the alveolar compartment [114]. Against this structural and functional background, the potential pulmonary effects of GLP-1/GLP-1R signalling may include preservation of alveolar type II cell homeostasis, support of epithelial regeneration after injury, and regulation of the pulmonary surfactant system [13, 113]. Experimental evidence further links GLP-1-related signalling to alveolar cell survival, bioenergetic regulation, and mitochondrial adaptation, together with attenuation of alveolar–capillary barrier disruption, pulmonary oedema, and overall lung injury [12, 115]. However, because the available evidence arises predominantly from non-infectious lung injury, metabolic respiratory disease, and animal models, infection studies using alveolar epithelial cell-specific GLP-1R manipulation will be required to distinguish cell-intrinsic pulmonary effects from systemic actions and to determine their relationship to pathogen control [32, 116]. Accordingly, assessment of GLP-1-related alveolar barrier protection should integrate SP-A and SP-B measurements, junctional-protein localisation, alveolar fluid clearance, pulmonary oedema, and gas-exchange function, with concordant structural and physiological readouts providing the strongest support for barrier restoration [112, 117].

At the vascular and microcirculatory interface, endothelial junctions, glycocalyx integrity, and microvascular perfusion collectively govern vascular permeability and tissue oxygenation [118, 119]. In severe infection and sepsis, reciprocal activation of inflammatory and coagulation pathways, together with oxidative stress and glycocalyx degradation, drives endothelial barrier failure, vascular leakage, and impaired organ perfusion [102, 120]. GLP-1RAs may influence vascular function by reducing oxidative stress, modulating endothelial inflammation, supporting nitric oxide signalling, and improving the metabolic milieu [5, 121]. Concurrent changes in glycaemia, body weight, blood pressure, and systemic inflammation may also contribute. These vascular effects should therefore be interpreted as a combination of local endothelial actions and systemic metabolic and inflammatory regulation [5, 121]. To determine whether these effects confer genuine endothelial and microcirculatory protection, circulating syndecan-1 and hyaluronic acid may serve as indicators of glycocalyx degradation, but changes in these biomarkers alone cannot establish structural restoration of the glycocalyx [122, 123]. More robust assessment requires integration of glycocalyx structure, endothelial junctional integrity—including VE-cadherin—vascular leakage, capillary perfusion, and organ function to establish whether endothelial barrier and microcirculatory recovery has occurred.

Across intestinal, pulmonary, and vascular compartments, GLP-1/GLP-1R-related actions may support host tolerance by preserving epithelial junctions and mucus-barrier integrity, maintaining alveolar epithelial and surfactant homeostasis, and protecting the endothelial glycocalyx and microcirculatory function [13, 107, 109]. However, this interpretation is based largely on metabolic and sterile injury models. Live-pathogen studies combining cell-type-specific GLP-1R manipulation with parallel assessment of pathogen burden and organ function are needed to determine whether barrier preservation complements, improves, or occurs independently of pathogen control [9, 116, 124].

### Autophagy and mitochondrial homeostasis

Autophagy and mitochondrial quality control integrate cell-autonomous antimicrobial defence, inflammatory regulation, and tissue repair after infection, thereby providing key homeostatic mechanisms that may influence both pathogen control and host tolerance [125, 126]. Selective autophagy recognises intracellular pathogens, microbial components, and damaged organelles and targets them for lysosomal degradation, thereby contributing to pathogen restriction, clearance of damage-associated molecular patterns, and innate immune regulation [127]. However, the consequences of autophagy during infection vary substantially with the pathogen, cellular context, and disease stage, because some viruses co-opt selective autophagy receptors or autophagic membranes to support replication, while intracellular bacteria may evade recognition, inhibit autophagosome maturation, or escape lysosomal degradation [128, 129]. Enhanced autophagic activity alone does not establish improved pathogen clearance, and its functional significance during infection requires parallel assessment of pathogen burden and cellular injury [130].

Evidence connecting GLP-1/GLP-1R signalling with the autophagy–lysosome system is currently derived predominantly from metabolic disease and sterile tissue-injury models [131]. Studies have associated GLP-1RA treatment with changes in LC3, Beclin-1, and p62, alongside reduced oxidative stress, improved cell survival, and less severe tissue injury [132]. These observations implicate autophagy in GLP-1-related tissue protection, but static marker changes alone cannot establish enhanced autophagic flux. LC3-II or autophagosome accumulation may indicate increased autophagosome formation but may equally reflect defective autophagosome–lysosome fusion or lysosomal degradation, while p62 abundance is shaped by both transcriptional regulation and protein turnover [133]. GLP-1-related autophagy during infection should be assessed using LC3 turnover in the presence and absence of lysosomal inhibition, dynamic p62 measurements, autophagosome–lysosome fusion, and lysosomal acidification and degradative capacity, rather than inferred from isolated measurements of LC3, Beclin-1, or p62 [134].

Mitochondrial homeostasis may constitute another key mechanism underlying GLP-1-related cytoprotection [135]. During infection, mitochondria support the energetic demands of immune effector responses and tissue repair while also functioning as signalling platforms for pathogen recognition, nucleic acid sensing, and the integration of inflammatory pathways [136]. Mitochondrial dysfunction is characterised by reduced oxidative phosphorylation and respiratory capacity, loss of membrane potential, accumulation of mitochondrial reactive oxygen species (mtROS), and release of mitochondrial DNA (mtDNA), accompanied by adaptive or pathological changes in mitochondrial fission, fusion, biogenesis, and mitophagy [137, 138]. Evidence from metabolic and sterile injury models links GLP-1RA treatment to improved mitochondrial architecture, bioenergetic function, and redox balance, thereby providing a mechanistic rationale for investigating its effects on mitochondrial injury during infection [139, 140]. Physiological amounts of mtROS may support phagocyte-mediated microbial killing and antiviral signalling, whereas sustained or excessive mtROS generation and cytosolic mtDNA accumulation can activate inflammasomes, cGAS–STING, and TLR9-associated pathways, promoting inflammatory cell death and tissue damage [141, 142]. Reductions in mtROS or mtDNA after GLP-1-related intervention should not be interpreted in isolation as evidence of benefit. Their functional significance should instead be evaluated alongside antimicrobial effector activity, pathogen burden, and tissue injury.

Available evidence supports autophagy and mitochondrial quality control as plausible mediators of GLP-1-related metabolic and cytoprotective effects, with a potential contribution to host tolerance during infection rather than an established role in direct pathogen restriction. However, because most evidence originates from non-infectious models and the reported phenotypes cannot yet be assigned to GLP-1R signalling intrinsic to infected or immune cells, cell-type-specific receptor manipulation will be required to separate direct cellular mechanisms from systemic effects [8]. Live-pathogen models with post-infection treatment are needed to assess autophagy–mitochondrial homeostasis, pathogen control, and tissue outcomes in parallel. Incorporating cell-type-specific Glp1r manipulation would help determine whether GLP-1-related interventions enhance host resistance, host tolerance, or both [100].

## Evidence across infectious diseases

**Fig. 2:**
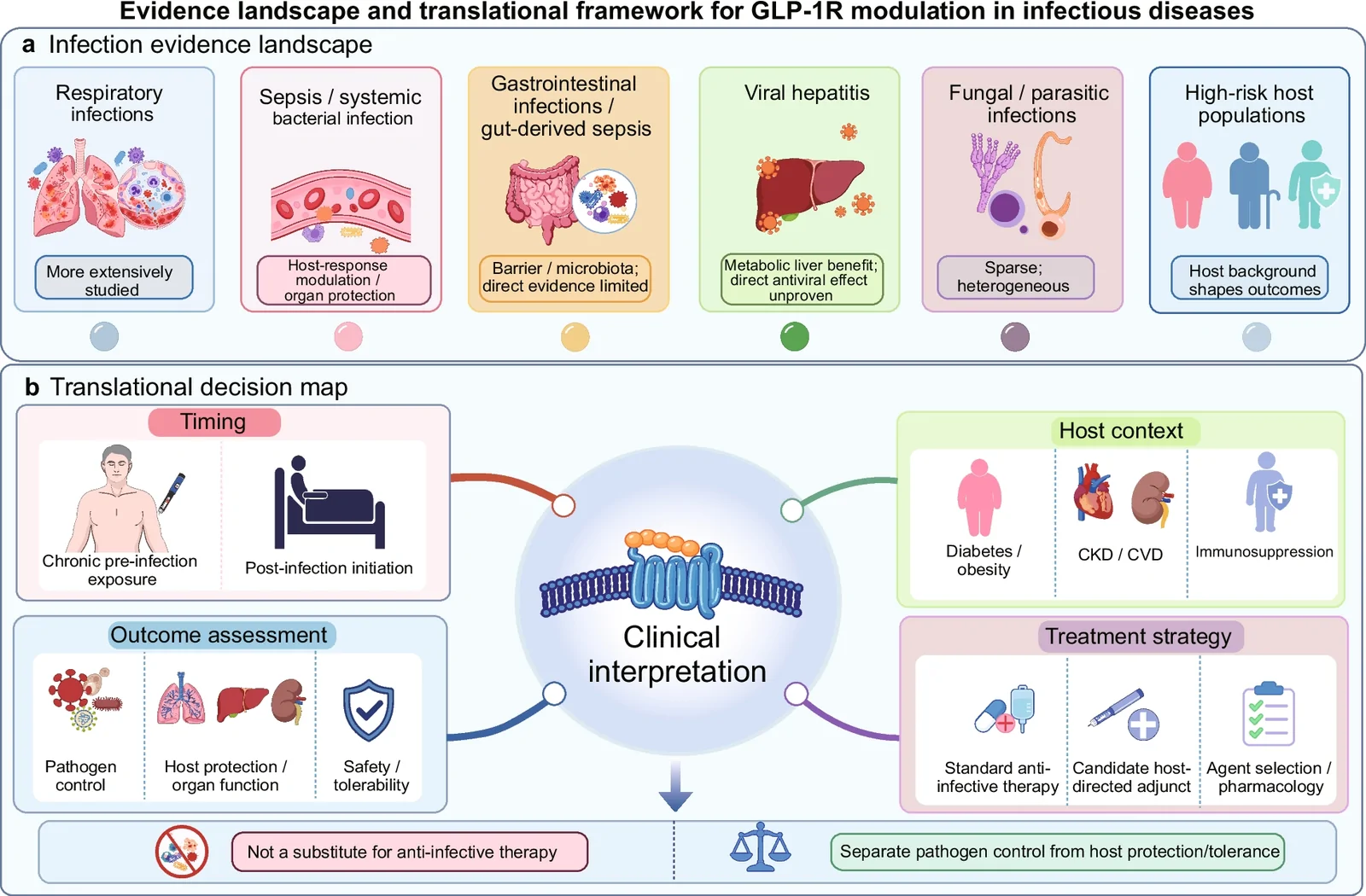
Evidence landscape and translational framework for GLP-1R modulation in infectious diseases. **a** Evidence across major infectious-disease contexts. Respiratory infections are comparatively well studied; sepsis and systemic bacterial infection mainly support host-response modulation and organ protection. Gastrointestinal infections and gut-derived sepsis are supported largely by barrier- and microbiota-related evidence, whereas direct infection-specific data remain limited. Evidence in viral hepatitis mainly concerns metabolic and hepatic outcomes, while fungal and parasitic evidence remains sparse. Evidence in high-risk host populations further highlights how host metabolic, cardiorenal, and immune status may modify infection-related outcomes. **b** Translational decision map integrating treatment timing, host context, outcome assessment, agent-specific pharmacology, and clinical use. Chronic pre-infection exposure should be distinguished from post-infection initiation, and pathogen control should be assessed separately from host protection or tolerance, organ function, and safety. GLP-1RAs should be considered candidate host-directed adjuncts rather than substitutes for standard anti-infective therapy. The figure was redrawn by the authors, with graphical elements adapted from BioGDP. Abbreviations: CKD, chronic kidney disease; CVD, cardiovascular disease; GLP-1R, glucagon-like peptide-1 receptor; GLP-1RA, glucagon-like peptide-1 receptor agonist

Building on the mechanisms outlined above, evidence linking GLP-1/GLP-1R signalling to infection can be organised into two complementary contexts. One classifies infections by pathogen type and predominantly affected organ system, whereas the other focuses on metabolically or medically high-risk hosts, including those with diabetes, obesity, chronic kidney disease, cardiovascular disease, or immunosuppression. The pathogen- and organ-based framework enables comparison of GLP-1-related effects across distinct pathogens and organ microenvironments, while the host-based framework highlights how baseline metabolic health, immune competence, and organ reserve may shape both infection outcomes and responses to GLP-1RA treatment (Fig. 2a). Within both contexts, the available evidence requires stratified interpretation according to the experimental infection model, the timing of intervention relative to infection, and the nature of the outcomes measured. Live-pathogen models permit integrated evaluation of pathogen control, host immune responses, and tissue injury, whereas endotoxaemia and sterile injury models primarily inform mechanisms of inflammatory modulation and organ protection and cannot independently establish effects on pathogen clearance [143, 144] (Table 2).

**Table 2:** Representative preclinical evidence in infection and infection-related models

Model and intervention	Pathogen outcome	Host outcome	Mechanistic interpretation and limitation	Reference(s)
*P. aeruginosa* Xen5 pneumonia–sepsis; liraglutide 2 mg/kg s.c., 30 min after infection	Reduced bacterial burden	Improved survival and surfactant indices; reduced lung injury	Supports both pathogen control and host tolerance; single strain, dose and short observation window	[13]
Diabetic *P. aeruginosa* pneumonia; Exendin-4 pretreatment for 7 days and continued dosing	Not measured	Reduced lung inflammation and pathology	Evidence in a metabolically high-risk host; pretreatment design and no burden/survival endpoint	[145]
*P. aeruginosa* airway mucus model; Exendin-4 during infection/post-infection protocols	Reduced lung bacterial burden	Improved FOXA2-related mucus homeostasis	More representative of chronic mucus-dysregulated airway disease than typical community-acquired pneumonia	[146]
CLP polymicrobial sepsis; liraglutide pretreatment	Not a principal endpoint	Improved endothelial function; reduced vascular inflammation and oxidative stress	Primarily supports endothelial and microcirculatory protection; prophylactic exposure	[147]
LPS-induced acute lung injury; dulaglutide treatment	Not applicable: no live pathogen	Reduced lung inflammation, oxidative stress and apoptosis	Supports an anti-inflammatory lung-protection mechanism only; LPS cannot establish pathogen clearance or infection efficacy	[11]
Caecal-slurry/hyperoxia two-hit lung injury; liraglutide pretreatment	No consistent reduction in pathogen burden	Reduced oedema, inflammation and alveolar–capillary injury	Predominantly supports host tolerance; composite model, prophylactic dosing and short follow-up	[12]
TLR challenge and polymicrobial sepsis; GLP-1R agonism with cell-specific Glp1r loss	Not a principal endpoint	Reduced TNF, sickness behaviour, hypothermia and lung injury	Supports a neuronal contribution to systemic host-response regulation; pathogen clearance was not established	[9]
PR8 H1N1 influenza; liraglutide from inoculation to day 2	Viral NP mRNA reduced; infectious titre not measured	Improved survival and interferon-related responses	Possible resistance signal, but viral-burden assessment was indirect	[116]
H9N2 influenza; liraglutide during the infection protocol	Viral titre unchanged	Reduced mortality, lung injury, and inflammatory indices	Predominantly supports host tolerance rather than antiviral activity	[148]
Experimental cerebral malaria; liraglutide from day 4 after infection	Parasitaemia unchanged	No meaningful survival or neurological benefit	Negative evidence against extrapolating benefit across pathogen classes	[149]

LPS models reproduce selected inflammatory components but do not contain replicating pathogens. The “Pathogen outcome” column distinguishes direct measures of pathogen burden/clearance from host-response outcomes. Abbreviations: *CLP* caecal ligation and puncture, *FOXA2* forkhead box A2, *LPS* lipopolysaccharide, *NP* nucleoprotein, *s.c.* subcutaneous, *TLR* Toll-like receptor, *TNF* tumour necrosis factor

### Respiratory infections

Respiratory infections currently constitute one of the most extensively studied settings for GLP-1/GLP-1R-related effects in infection, although the evidential basis and the conclusions that can be drawn differ substantially among bacterial pneumonia, influenza, and COVID-19. For bacterial pneumonia and lower respiratory tract infection, several live-bacterial animal studies—most involving *Pseudomonas aeruginosa*—are available, but variation in the assessment of bacterial burden, survival, and pulmonary pathology limits direct comparison and integrated interpretation [13, 145, 146]. By contrast, the influenza literature is based largely on mouse models of a limited number of viral subtypes, such as H9N2, with most studies emphasising pulmonary inflammation, acute lung injury, and overall disease severity rather than comprehensive assessment of viral control [116, 148]. Human evidence for COVID-19 differs further, consisting predominantly of observational analyses and evidence syntheses of pre-existing GLP-1RA exposure, with no comparable evidence from randomised controlled trials initiating treatment after a confirmed diagnosis of infection [150]. Interpretation across respiratory infections therefore requires parallel but distinct evaluation of host resistance—through pathogen burden, replication, and clearance—and host tolerance—through pulmonary inflammation, barrier integrity, alveolar function, and organ injury [100].

#### Bacterial pneumonia

The effects of GLP-1/GLP-1R-related interventions in bacterial pneumonia cannot be inferred from reduced pulmonary inflammation alone and require parallel assessment of bacterial burden, survival, lung function, and tissue injury. The most direct evidence derives from a murine model of pneumonia-associated sepsis established by intratracheal inoculation with bioluminescent *Pseudomonas aeruginosa* Xen5, followed by subcutaneous liraglutide at 2 mg/kg 30 min after infection as an early therapeutic intervention. Liraglutide was associated with lower in vivo bacterial burden, improved survival, reduced pulmonary inflammation and tissue injury, and increased SP-A, SP-B, and surfactant phospholipid levels [13]. By integrating live bacterial burden, survival, and pulmonary pathology, this study provides more direct evidence of infection-specific activity than endotoxin-based lung injury models. Nevertheless, interpretation is constrained by the use of one bacterial strain, one liraglutide dose, and a short follow-up period, while cell-specific Glp1r manipulation has not yet defined receptor dependence or distinguished alveolar epithelial from immune-cell-mediated effects [13].

Two further studies using live *P. aeruginosa* models indicate that GLP-1RA-associated effects may vary according to host metabolic status, airway pathology, and the timing of administration. In high-fat-diet- and streptozotocin-induced diabetic rats, Exendin-4 was administered for 7 days before infection and continued after challenge [145]. Treatment was associated with reduced pulmonary inflammation, lower TNF-α, IL-1β, and IL-6 expression, and attenuation of related signalling pathways [145]. Because bacterial burden and survival were not measured, these findings support inflammatory modulation in a metabolically high-risk host but do not establish improved pathogen control or survival. A separate study linked Exendin-4 to restoration of FOXA2-associated airway mucus homeostasis and reduced lung bacterial burden after *P. aeruginosa* PAO1 infection, with a similar effect observed when pyocyanin pre-exposure preceded infection and treatment was initiated thereafter [146]. These findings directly link improved mucus homeostasis with lower bacterial burden, but the airway pathology more closely models infection associated with cystic fibrosis or chronic obstructive pulmonary disease than conventional acute community-acquired pneumonia.

Endotoxin-induced acute lung injury models provide complementary evidence for the anti-inflammatory and barrier-protective potential of GLP-1RAs. In LPS-induced lung-injury models, GLP-1R-related interventions have been associated with reduced pulmonary inflammation, oedema, apoptosis, and endothelial-barrier disruption [11, 151]. Complementary in vitro evidence shows that Exendin-4 suppresses LPS-induced inflammatory and oxidative responses in RAW264.7 macrophages [152]. Dulaglutide reduced inflammatory infiltration, cytokine responses, and apoptosis-related readouts in LPS-induced lung injury, while liraglutide pretreatment attenuated pulmonary inflammation, oedema, and alveolar–capillary damage and improved short-term survival in a caecal-slurry sepsis model combined with hyperoxia [11, 12]. As endotoxin models lack replicating bacteria and the sepsis–hyperoxia study used prophylactic pretreatment within a composite injury setting, these findings principally inform lung protection and host tolerance rather than pathogen clearance in primary bacterial pneumonia.

Live-pathogen studies suggest that GLP-1RAs may affect both bacterial control and pulmonary injury, but the evidence is dominated by *P. aeruginosa* models and is heterogeneous with respect to host phenotype, intervention timing, and outcome selection. More specifically, early therapeutic liraglutide was associated with improvements in bacterial burden, survival, and lung pathology, whereas the diabetic model primarily informs inflammatory regulation and the mucus-dysfunction model links restoration of airway homeostasis with lower bacterial burden [13, 145, 146]. Evidence beyond *P. aeruginosa* is needed, particularly for clinically important pathogens such as Streptococcus pneumoniae and Staphylococcus aureus. Studies initiating GLP-1RA treatment after a clinically relevant delay and alongside standard antibiotics could follow pathogen clearance, pulmonary function, and survival longitudinally. Cell-specific Glp1r manipulation would help distinguish effects on host resistance from those on host tolerance**.**

#### Influenza virus infection

Direct evidence for GLP-1/GLP-1R-related intervention in influenza is currently limited to two live-virus mouse studies that differed substantially in viral subtype, experimental design, and assessment of pathogen-related outcomes. In one study, male mice were infected intratracheally with A/Puerto Rico/8/34 (H1N1; PR8), and infection outcomes were examined following liraglutide treatment or global Glp1r deletion. Subcutaneous liraglutide administered from the day of inoculation through post-infection day 2 improved survival and reduced pulmonary influenza nucleoprotein (NP) mRNA, accompanied by increased expression of several interferon-inducible GTPases [116]. As NP mRNA provides only an indirect estimate of viral burden, these findings offer a preliminary signal of altered viral control rather than definitive evidence and should be validated by measuring infectious viral titres and longitudinal clearance kinetics. Notably, global Glp1r deletion did not significantly affect NP expression, body-weight loss, or survival after infection, revealing a lack of full concordance between pharmacological agonism and receptor deficiency and leaving the receptor dependence and principal tissue mediators of liraglutide protection unresolved [116].

A second study established influenza-associated acute lung injury in mice using live A/Chicken/Hebei/4/2008 (H9N2) virus. Liraglutide reduced weight loss, anorexia, and mortality, as well as the lung index, inflammatory-cell infiltration, bronchoalveolar lavage fluid protein, and pulmonary IL-1β, IL-6, and TNF-α [148]. Despite these improvements, lung viral titres were unchanged. This pattern suggests that the benefit primarily reflected reduced pulmonary inflammation and tissue injury rather than suppression of H9N2 replication [148]. Together, these studies indicate that the effects of liraglutide in influenza may be contingent on viral strain and experimental context. In the PR8 model, improved survival coincided with lower NP mRNA, whereas in the H9N2 model, survival and lung injury improved despite unchanged viral titres [116, 148]. Although the current evidence includes a preliminary signal of altered host resistance, it more consistently supports attenuation of pulmonary immunopathology and enhancement of host tolerance.

Across H1N1 and H9N2 mouse models, liraglutide has been associated with improved survival and reduced lung injury, whereas evidence for effects on viral replication and clearance remains inconsistent [116, 148]. At this stage, GLP-1RAs should be regarded as candidate host-directed therapies rather than as established direct antiviral agents [8]. More informative studies would initiate GLP-1RA treatment at clinically relevant intervals after infection and assess infectious viral titres, clearance kinetics, pulmonary function, and survival alongside standard antiviral therapy. Tissue- or cell-specific Glp1r manipulation would then help distinguish effects on host resistance from those on host tolerance.

#### SARS-CoV-2 infection and COVID-19

COVID-19 and metabolic dysfunction are linked through a bidirectional relationship [153]. Diabetes, obesity, and chronic kidney disease predispose patients to severe COVID-19, mechanical ventilation, and death, while SARS-CoV-2 infection and its systemic stress response may, in turn, precipitate hyperglycaemia, exacerbate insulin resistance, and contribute to new-onset diabetes [154–156]. Outcomes in severe COVID-19 are further shaped by alveolar and vascular endothelial injury, immunothrombosis, and progressive multiple-organ dysfunction [157].

Current human evidence largely reflects pre-existing GLP-1RA exposure before SARS-CoV-2 infection, rather than therapeutic initiation after COVID-19 diagnosis. In an observational analysis from the National COVID Cohort Collaborative, pre-infection GLP-1RA use in patients with type 2 diabetes was associated with reduced 60-day mortality and lower risks of emergency department attendance and hospitalisation. The adjusted odds of death within 60 days were also lower among GLP-1RA users than among those receiving DPP-4 inhibitors [158]. Meta-analytic evidence from observational studies similarly associates pre-existing GLP-1RA use with lower COVID-19 mortality [159]. These observations support a potential protective signal from long-term GLP-1RA exposure before infection, but residual confounding related to baseline metabolic health, obesity severity, concomitant treatment, prescribing selection, and healthcare utilisation remains important.

Secondary analyses of large randomised cardiovascular and kidney outcome trials provide a complementary source of evidence on sustained GLP-1RA exposure beyond observational cohorts. SELECT randomised participants with overweight or obesity and established cardiovascular disease, but without diabetes, to semaglutide 2.4 mg or placebo. Although semaglutide did not reduce COVID-19 incidence, it was associated with fewer COVID-19-related serious adverse events and deaths, while the reduction in non-cardiovascular mortality was driven largely by fewer infection-related deaths [160]. In FLOW, sustained semaglutide 1.0 mg treatment among patients with type 2 diabetes and chronic kidney disease was associated with fewer serious infectious adverse events, infection-related hospitalisations, and COVID-19-related events [161]. Together, these results suggest that sustained pre-infection GLP-1RA exposure may be associated with more favourable COVID-19 outcomes in selected metabolically or cardiorenally high-risk populations. Because SELECT and FLOW were designed primarily around cardiovascular and kidney outcomes and treatment preceded infection, their COVID-19 findings are best interpreted as signals of long-term optimisation of metabolic health, cardiorenal reserve, and host tolerance rather than evidence of acute anti-infective efficacy.

Evidence for initiating GLP-1RAs after COVID-19 diagnosis is limited but continues to evolve. Observational evidence on prior use and long-term randomised exposure in SELECT and FLOW provides a rationale for acute-phase trials, but dedicated studies are needed to determine the efficacy, safety, and optimal timing of GLP-1RA initiation after confirmed infection [158, 160, 161]. Because acute COVID-19 may involve reduced intake, nausea, vomiting, dehydration, haemodynamic instability, and fluctuating kidney function, future trials should integrate virological endpoints with respiratory-support requirements, organ function, clinical recovery, and gastrointestinal and renal safety [162, 163]. Current evidence therefore positions GLP-1RAs primarily within long-term metabolic and organ-risk management; their role as host-directed therapies during acute COVID-19 has yet to be established. Future trials should prospectively initiate treatment after diagnosis, stratify patients by treatment timing and disease severity, and evaluate GLP-1RAs alongside standard antiviral and supportive care.

Three interrelated host-directed mechanisms may help explain the association between sustained GLP-1RA use and more favourable COVID-19 outcomes. First, sustained improvements in glycaemic control, insulin sensitivity, body weight, and chronic metabolic inflammation may reduce pre-existing immune dysfunction and organ susceptibility before infection [164, 165]. Second, enhanced cardiorenal reserve, endothelial function, and microcirculatory integrity may reduce susceptibility to the endothelial injury and immunothrombosis characteristic of severe COVID-19 [157, 166, 167]. Third, GLP-1R-associated restraint of inflammation and potential central neuroimmune regulation may limit host-mediated tissue injury; however, evidence for neuronal GLP-1R dependence comes primarily from Toll-like receptor challenge and polymicrobial sepsis models and cannot yet be directly extrapolated to COVID-19 [9]. Proposed effects of SARS-CoV-2 on GLP-1R transcription, membrane trafficking, receptor internalisation, or endoplasmic-reticulum-stress signalling should currently be considered testable hypotheses rather than established mechanisms.

Overall, available clinical evidence associates sustained pre-infection GLP-1RA use with fewer severe COVID-19 events or deaths in selected metabolically and cardiorenally high-risk populations [159–161]. These findings are more consistent with long-term metabolic risk reduction, preservation of organ reserve, and enhanced host tolerance than with a direct antiviral effect. Whether GLP-1RA treatment initiated during acute COVID-19 modifies viral control, host tolerance, or both remains unresolved.

The respiratory infection literature is characterised by substantial heterogeneity in pathogen, experimental model, and study design. Limited live-bacterial studies suggest that GLP-1RAs may influence both pathogen control and protection against pulmonary injury [13]. Influenza studies more consistently indicate reduced pulmonary immunopathology and enhanced host tolerance, while evidence for altered viral replication or clearance remains model-dependent [116, 148]. COVID-19 evidence, by contrast, is dominated by observational studies of pre-existing use and secondary analyses of long-term randomised metabolic trials, supporting metabolic risk reduction and preservation of organ reserve rather than efficacy of treatment initiated after diagnosis [159, 160]. Across respiratory infections, the most consistent evidence concerns host-response modulation and limitation of pulmonary injury, whereas effects on pathogen clearance remain infection- and model-specific [100]. Because severe respiratory infection may progress from local lung injury to systemic inflammation and multiple-organ dysfunction, the relevance of GLP-1/GLP-1R signalling must next be considered in sepsis and systemic bacterial infection.

### Sepsis and systemic bacterial infection

Sepsis is defined as life-threatening organ dysfunction resulting from a dysregulated host response to infection and encompasses pathogen expansion, inflammatory dysregulation, endothelial and microcirculatory injury, metabolic stress, and the potential development of subsequent immunosuppression [88, 168]. Interpretation of GLP-1/GLP-1R-related evidence requires clear separation of LPS endotoxaemia, which lacks replicating pathogens, from polymicrobial infection models such as caecal ligation and puncture (CLP) or caecal slurry and from defined live-bacterial models that quantify pathogen burden in blood and tissues [144, 169]. LPS models principally inform inflammatory regulation, whereas polymicrobial models capture systemic inflammation and organ injury [100, 170, 171]. Conclusions regarding host resistance require quantitative pathogen-burden and clearance data. Defined live-bacterial models are needed to determine whether GLP-1-related interventions influence pathogen control, host tolerance, or both [100, 170, 171].

Systemic inflammation may itself induce an endogenous GLP-1 response. Endotoxin, IL-1β, and IL-6 can stimulate GLP-1 secretion, with the endotoxin-induced response depending on IL-6 and coinciding with increased insulin and lower blood glucose, consistent with a potential role in metabolic adaptation to infectious stress [172]. Clinically, early or sustained elevations in circulating GLP-1 have been associated with mortality, persistent organ dysfunction, and unfavourable long-term outcomes in sepsis, potentially serving as a marker of ongoing inflammation and unresolved catabolism rather than a protective response alone [173]. Evidence from polymicrobial models predominantly supports vascular and organ protection by GLP-1RAs during sepsis [147]. Because liraglutide was administered prophylactically before CLP and the study focused on vascular function, oxidative stress, and early inflammation without longitudinal assessment of bacterial clearance, its findings principally support endothelial and microcirculatory protection rather than improved pathogen control or long-term survival [147]. In a double-hit model combining caecal slurry-induced sepsis with 95% hyperoxia, 3 days of liraglutide pretreatment reduced disease severity and improved short-term survival while attenuating pulmonary inflammation, oedema, alveolar–capillary barrier injury, and extrapulmonary organ damage [12]. Because pathogen burdens did not improve consistently across the lungs and spleen, the overall findings are more readily interpreted as enhanced pulmonary tolerance and multiorgan protection than as a general improvement in pathogen clearance [12]. In caecal-slurry sepsis, neuronal Glp1r deletion substantially diminished the beneficial effects of GLP-1R agonism on sickness behaviour, hypothermia, systemic inflammation, and lung injury, whereas comparable deletion in haematopoietic or endothelial cells did not [9]. These findings implicate central neuronal GLP-1R in the regulation of sepsis-associated host responses and organ injury, but the emphasis on inflammatory and tissue-injury endpoints precludes conclusions regarding improved pathogen clearance.

In contrast to these polymicrobial studies, live-bacterial pneumonia provides an initial signal that GLP-1RAs may also affect host resistance. In *Pseudomonas aeruginosa* pneumonia-associated sepsis, liraglutide initiated 30 min after infection was associated with lower bacterial burden, improved survival, reduced pulmonary inflammation and injury, and restoration of surfactant-related readouts [13]. These results suggest that liraglutide may simultaneously enhance local pathogen control and pulmonary tolerance in this infection setting, but whether preservation of alveolar epithelial and surfactant homeostasis is required and GLP-1R-dependent has not been resolved. The apparent antibacterial signal is limited by the use of a single strain and short follow-up and should be reproduced with other clinically relevant pathogens alongside standard antibiotics, fluid resuscitation, and source control.

Available clinical evidence predominantly concerns whether sustained GLP-1RA exposure before infection modifies subsequent infection risk. Real-world analyses associate GLP-1RA use with a lower incidence of severe sepsis than DPP-4 inhibitor treatment, and systematic evidence from randomised trials similarly suggests possible reductions in overall and severe infections [14, 41]. These associations are best interpreted as signals of long-term metabolic and host-risk modification rather than evidence of acute sepsis treatment, with study design, residual confounding, and variation among high-risk populations should also be considered. Dedicated therapeutic studies must therefore determine whether GLP-1RA initiation after a diagnosis of sepsis alters pathogen clearance, organ dysfunction, or survival.

Current evidence most consistently supports modulation of early systemic inflammation and protection of the vascular endothelium, microcirculation, lungs, and other vulnerable organs during sepsis. CLP and caecal-slurry models predominantly indicate enhanced host tolerance, whereas limited live-bacterial pneumonia evidence raises the possibility of concurrent improvement in local pathogen control. Studies should now initiate treatment at defined intervals after infection on a background of standard fluid resuscitation, antibiotics, and source control, with longitudinal assessment of bacteraemia, blood and tissue bacterial burdens, clearance kinetics, organ function, survival, immunosuppression, and secondary infection.

### Gastrointestinal infections and gut-derived sepsis

In contrast to respiratory infection and systemic sepsis, direct evidence linking GLP-1/GLP-1R signalling to gastrointestinal infection remains sparse. Most of the available evidence derives from anti-CD3-induced T-cell inflammation, dextran sulphate sodium (DSS) colitis, germ-free mice, and caecal ligation and puncture (CLP) sepsis rather than from infectious enteritis models involving defined live gastrointestinal pathogens [49, 174–176]. Consequently, existing studies mainly inform mucosal immune regulation, colonic homeostasis, microbiota changes, and tissue injury and do not yet establish effects on intestinal pathogen colonisation, clearance, or extraintestinal dissemination. Interpretation must further distinguish pathogen colonisation and invasion at the intestinal mucosa from CLP-induced polymicrobial peritonitis and systemic sepsis caused by leakage of intestinal contents into the peritoneal cavity.

Intestinal intraepithelial lymphocytes represent one of the few cell populations for which a functional link between GLP-1R signalling and intestinal immunity is relatively well supported. Early work identified relative enrichment of *Glp1r* transcripts in murine intestinal intraepithelial lymphocyte preparations and demonstrated that Exendin-4 elicited cAMP responses and reduced activation-induced inflammatory mediator production in purified cells, with diminished effects after *Glp1r* deletion, providing functional support for local receptor-dependent immune regulation [177]. Subsequent cell-specific genetic studies showed that intraepithelial lymphocyte GLP-1R was dispensable for the systemic metabolic effects of GLP-1RAs [174]. By contrast, intraepithelial lymphocyte GLP-1R contributed to changes in gut microbial composition and to modulation of anti-CD3-induced intestinal and systemic T-cell inflammation, whereas GLP-1RA effects during LPS challenge did not require GLP-1R in these cells [174]. Together, these studies support a functional and genetically defined connection between enteroendocrine GLP-1 signalling and mucosal immunity; however, because the models predominantly capture T-cell-driven inflammation, they do not establish effects on the colonisation, tissue invasion, or clearance of live gastrointestinal pathogens.

More recent non-infectious studies extend this evidence to the maintenance of colonic tissue homeostasis. In DSS colitis, GLP-1RAs reduced weight loss, colon shortening, and histopathological damage while increasing IL-22 production by group 3 innate lymphoid cells (ILC3s)**,** and the attenuation of protection in ILC3-deficient animals implicated IL-22⁺ ILC3s in the response to chemically induced colonic injury [175]. Germ-free GLP-1R-deficient mice developed colonic cellular energy deficiency, endoplasmic reticulum stress, and mitochondrial abnormalities together with goblet-cell loss, increased caecal water content, and impaired epithelial integrity, phenotypes that were partly corrected by increased dietary energy availability or recolonisation with a conventional microbiota [49]. Collectively, these findings connect GLP-1R signalling with mucosal inflammatory regulation, goblet-cell maintenance, and colonic homeostasis, but their significance for gastrointestinal infection remains to be established in defined live-pathogen challenge models.

The caecal ligation and puncture (CLP) model provides a more infection-relevant setting in which to examine GLP-1RA-associated effects during gut-derived polymicrobial sepsis. In a 2026 study, liraglutide pretreatment before CLP improved survival and reduced systemic inflammation as well as colonic, pulmonary, and hepatic injury [176]. Treatment also remodelled the gut microbiota and faecal metabolome. Antibiotic-mediated microbiota depletion attenuated the survival benefit, whereas faecal microbiota transfer from liraglutide-treated donors reproduced part of the survival and organ-protective phenotype [176]. These intervention experiments provide functional support for a microbiota-mediated component of liraglutide-associated host protection; however, the prophylactic design and emphasis on survival, inflammation, histopathology, microbial composition, and metabolomic changes limit conclusions regarding pathogen control and intestinal barrier function. Bacterial burdens were not systematically quantified in peritoneal fluid, mesenteric lymph nodes, blood, liver, or spleen, and functional permeability, junctional-protein localisation, and bacterial translocation were not assessed as principal endpoints [176]. The observed microbiota and tissue phenotypes therefore support ecological remodelling, enhanced host tolerance, and multiorgan protection rather than established bacterial clearance. Improved pathogen control would require direct quantification of bacterial burden and clearance, alongside assessment of translocation and functional barrier integrity.

No clinical studies have yet established the effects of initiating GLP-1RA therapy during acute infectious enteritis. GLP-1RAs slow gastric emptying and commonly produce gastrointestinal effects such as nausea, vomiting, diarrhoea, constipation, and early satiety, with their frequency and severity varying according to the agent, dose, treatment phase, and baseline gastrointestinal motility [110]. Delayed gastric emptying may further modify the rate of absorption and time to peak concentration of selected oral medicines, although effects on total systemic exposure vary between drugs [178]. In patients with persistent vomiting or diarrhoea, poor oral intake, dehydration, or a requirement for reliable exposure to oral anti-infective therapy, decisions regarding GLP-1RA continuation or initiation during acute infection must be individualised according to clinical status and treatment priorities.

Taken together, current evidence links GLP-1/GLP-1R signalling to mucosal immune regulation involving intestinal intraepithelial lymphocytes and IL-22⁺ group 3 innate lymphoid cells, as well as to goblet-cell maintenance, colonic metabolic homeostasis, microbiota remodelling, and organ protection in CLP sepsis [49, 174–176]. At present, the evidence more strongly supports barrier preservation and host tolerance than enhanced host resistance, and direct effects on pathogen-specific enteritis, intestinal pathogen clearance, and extraintestinal dissemination remain unproven. Future work should use defined live enteric pathogens and initiate treatment after infection. Studies should measure faecal shedding, intestinal and bloodstream pathogen burdens, and translocation to mesenteric lymph nodes and distant organs in parallel with functional permeability, tight-junction localisation, mucus-layer integrity, organ injury, and survival. This design would separate effects on host resistance from those on host tolerance.

### Viral hepatitis

Direct evidence for a role of GLP-1/GLP-1R signalling in viral hepatitis is sparse, with current research focusing mainly on whether GLP-1RAs can mitigate additional metabolic liver injury in patients with hepatitis B virus (HBV) or hepatitis C virus (HCV) infection and coexisting diabetes, obesity, or metabolic dysfunction-associated steatotic liver disease (MASLD). Studies directly assessing viral replication are largely confined to HCV cell-culture systems [179]. By contrast, clinical investigations have focused mainly on long-term hepatic outcomes rather than direct virological endpoints [180]. Accordingly, metabolic-comorbidity-related hepatoprotection must be distinguished from infection-specific effects on viral replication.

Host metabolic status is an important modifier of chronic viral hepatitis progression. HCV infection is closely linked to insulin resistance, type 2 diabetes, hepatic steatosis, and dysregulated lipid metabolism, all of which may accelerate fibrosis progression and increase the burden of extrahepatic complications [181]. The relationship between chronic HBV infection and metabolic dysfunction is more heterogeneous and varies according to age, adiposity, virological status, and the stage of liver disease. In patients with chronic hepatitis B treated with nucleos(t)ide analogues, diabetes is associated with higher risks of cirrhosis, hepatocellular carcinoma, and mortality, while clustering of multiple metabolic disorders further predicts unfavourable long-term outcomes [182, 183]. These findings establish metabolic dysfunction as a host modifier of viral hepatitis progression but do not demonstrate that metabolic improvement translates into reduced viral replication, which requires direct virological assessment.

Evidence specifically addressing GLP-1RAs in viral hepatitis is currently limited to an in vitro HCV study and an observational cohort in chronic hepatitis B. In Huh-7.5 cells infected with JFH-1 HCV, liraglutide reduced HCV RNA and NS5B protein, and these effects were attenuated by pharmacological AMPK inhibition or gene silencing, providing an initial experimental signal that GLP-1RA treatment may influence HCV replication [179]. However, the use of a hepatoma cell line, high drug concentrations, and a single viral culture system limits extrapolation, and both in vivo reproducibility and dependence on canonical GLP-1R signalling remain to be established. In contrast, a nationwide new-user cohort of patients with chronic hepatitis B and type 2 diabetes found broadly comparable risks of hepatocellular carcinoma, cirrhosis, liver transplantation, and liver-related mortality among initiators of GLP-1RAs, SGLT2 inhibitors, and DPP-4 inhibitors [180]. Although this cohort informs long-term hepatic outcomes in patients with chronic hepatitis B and diabetes, the absence of HBV DNA, HBsAg kinetics, and serological-conversion endpoints means that the findings cannot be interpreted as evidence of anti-HBV activity.

Randomised GLP-1RA trials in MASLD/metabolic dysfunction-associated steatohepatitis (MASH) offer indirect evidence for patients with viral hepatitis and metabolic comorbidity, but treatment effects vary across stages of metabolic liver disease. In non-cirrhotic NASH, phase II evidence showed that semaglutide increased steatohepatitis resolution, but did not produce a statistically significant improvement in fibrosis stage [184]. In MASH-related compensated cirrhosis, semaglutide did not significantly improve fibrosis or steatohepatitis resolution [185]. Subsequent phase III evidence demonstrated that weekly semaglutide improved both MASH resolution and fibrosis outcomes in patients with F2–F3 fibrosis [186]. These trials demonstrate stage-dependent improvements in weight, glycaemic control, and liver histology in metabolic liver disease; however, because neither the populations nor the endpoints addressed HBV or HCV infection, the findings support mitigation of metabolic liver injury rather than improved viral control.

In patients with viral hepatitis complicated by diabetes, obesity, or MASLD, GLP-1RAs may therefore have a role in managing metabolic comorbidity, principally through reductions in insulin resistance, hepatic lipid accumulation, and metabolic liver injury rather than through an established direct antiviral effect [179, 187, 188]. Key next steps are to assess viral control and host tissue protection in parallel on a background of standard antiviral therapy. HBV studies should incorporate HBV DNA, quantitative HBsAg, HBeAg seroconversion, and virological breakthrough, while HCV studies should assess HCV RNA kinetics and sustained virological response, with concurrent evaluation of hepatocellular injury, steatosis, inflammation, fibrosis, decompensation, and hepatocellular carcinoma [189–191]. Mechanistic studies should combine receptor blockade with cell-specific Glp1r manipulation to separate direct effects in infected hepatocytes or intrahepatic immune populations from indirect effects arising through weight loss, improved glycaemia, and broader systemic metabolic changes [8].

### Fungal and parasitic infections

In contrast to bacterial pneumonia, sepsis, and selected viral infections, direct evidence linking GLP-1/GLP-1R signalling to fungal and parasitic infections remains sparse. Current evidence is derived mainly from a hyperglycaemic mouse model of cutaneous *Candida albicans* infection, experimental cerebral malaria, and observational analyses of endogenous incretin profiles in individuals with helminth infections [149, 192–194]. These studies are highly heterogeneous with respect to pathogen, host metabolic background, manipulation of GLP-1 signalling, and outcome selection. Thus, interpretation must distinguish indirect benefits arising from improved glycaemic control and reduced metabolic stress, infection-associated changes in endogenous GLP-1, and direct GLP-1R-dependent effects on pathogen control.

Diabetes and inadequately controlled hyperglycaemia increase susceptibility to *Candida* colonisation and infection, potentially by altering local glucose availability and impairing phagocyte-mediated antifungal defence, but these associations do not establish a specific role for GLP-1 signalling in antifungal immunity [195]. In a 2026 preprint using hyperglycaemic mice with cutaneous *C. albicans* infection, liraglutide was associated with smaller lesions, lower tissue fungal burden, and reduced inflammation and tissue damage, raising the possibility that metabolic improvement may restore antifungal defence compromised by diabetes [192]. These findings provide an initial signal that GLP-1RA treatment may improve both fungal control and tissue tolerance; however, confirmation in independent models using glycaemia-matched controls, GLP-1R blockade, and cell-specific Glp1r manipulation is required to separate indirect metabolic effects from direct receptor-dependent mechanisms.

Complementary work shows that *Candida auris* induces macrophage glycolysis without generating an effective IL-1β response or limiting fungal growth [196]. This finding indicates that increased immune-cell metabolism does not necessarily improve antifungal clearance, but it does not provide direct evidence of GLP-1RA activity because GLP-1 signalling was not manipulated in the study [196].

The clearest interventional evidence in parasitic infection derives from experimental cerebral malaria. In *Plasmodium berghei* ANKA-infected mice, liraglutide initiated on post-infection day 4 neither altered the development or progression of cerebral malaria nor improved survival, while peripheral parasitaemia was unchanged or increased at selected time points [149]. The same study also found no clear antiparasitic activity in cultured *Plasmodium falciparum.* Thus, neuroprotection reported in sterile neurological injury models did not translate into an infectious benefit in experimental cerebral malaria, and no direct antiplasmodial effect was demonstrated.

Helminth studies have focused mainly on associations between infection status, endogenous incretin profiles, and metabolic phenotypes. In non-diabetic individuals with obesity, *Strongyloides stercoralis* infection was associated with lower circulating GLP-1, insulin, glucagon, and C-peptide but higher GIP, suggesting that chronic helminth infection can reshape the host nutritional–incretin milieu [193]. Conversely, in individuals with type 2 diabetes, hookworm infection was associated with higher GLP-1 and GIP levels, which decreased following anthelmintic therapy [194]. Because GLP-1R was neither activated nor inhibited and parasite burden, expulsion, and protection against reinfection were not evaluated, these studies establish only context-dependent associations between helminth infection and the host metabolic–incretin network.

Taken together, the available evidence does not establish direct or broadly applicable antifungal or antiparasitic activity of GLP-1RAs. A single preprint in hyperglycaemic mice with cutaneous *C. albicans* infection reported lower fungal burden together with reduced inflammation and tissue injury [192]. By contrast, liraglutide did not improve parasitaemia or survival in experimental cerebral malaria [149], and the available helminth studies are observational [193, 194]. Future studies should use defined live-pathogen models, post-infection treatment, standard antifungal or antiparasitic therapy, and parallel assessment of pathogen burden, immune responses, tissue injury, and survival to distinguish pathogen control from metabolic correction and host tolerance.

### Infection in high-risk host populations

Whereas the preceding discussion organised the evidence by pathogen and predominantly affected organ, this section examines infection through the complementary perspective of host susceptibility, focusing on diabetes, obesity, chronic kidney disease, cardiovascular disease, and iatrogenic immunosuppression. These populations are predisposed to infection and severe disease through varying combinations of metabolic dysregulation, immune dysfunction, and reduced organ reserve, although the dominant mechanisms differ among high-risk host groups. Available clinical evidence predominantly reflects sustained GLP-1RA exposure before infection and assesses incident infections, infection-related hospitalisation, or mortality rather than treatment efficacy after an infection has been diagnosed.

Diabetes and obesity constitute the most extensively studied high-risk host backgrounds. Sustained hyperglycaemia and obesity-associated metabolic inflammation disrupt both innate and adaptive immunity and may thereby increase susceptibility to, or the severity of, selected bacterial and viral infections [82, 197–199]. In an active-comparator, new-user real-world analysis, patients with type 2 diabetes initiating GLP-1RAs had lower risks of pneumonia and severe sepsis than those initiating DPP-4 inhibitors, with a risk ratio of 0.61 for severe sepsis (95% CI 0.59–0.63) [14]. Systematic evidence from randomised trials similarly suggests reductions in overall, severe, and selected respiratory infections, but these events were typically captured as safety or secondary outcomes in trials designed around metabolic, weight-management, or cardiovascular endpoints [41]. These findings are consistent with the possibility that sustained improvements in glycaemic control, weight, and metabolic risk reduce susceptibility to selected infections, although residual confounding related to baseline health, treatment selection, adherence, and co-therapy cannot be excluded.

Observational cohorts and secondary analyses of SELECT and FLOW associate sustained GLP-1RA exposure with fewer severe infection-related outcomes in selected metabolically or cardiorenally high-risk populations [41, 160, 161]. These findings most likely reflect long-term optimisation of metabolic health, cardiorenal reserve, and host tolerance rather than acute pathogen-directed efficacy, and they do not establish benefit from treatment initiated after infection diagnosis.

In contrast, evidence in iatrogenically immunosuppressed and haematopoietic stem-cell transplantation populations remains preliminary. In paediatric allogeneic haematopoietic stem-cell transplantation, circulating endogenous GLP-1 correlated with systemic inflammatory and mucosal-injury markers, providing observational evidence linking the incretin response to post-transplant mucosal stress [200]. As GLP-1 signalling was not therapeutically manipulated, these findings identify a biomarker association rather than an effect on infection outcomes or treatment efficacy. An ongoing randomised, double-blind trial is testing semaglutide for gastrointestinal mucositis after high-dose chemotherapy and autologous transplantation, but the available publication is limited to the protocol and provides no efficacy or infection-specific outcome data [201]. Experimental acute graft-versus-host disease provides an important counterpoint to the potentially favourable findings from other settings. Semaglutide had limited effects on systemic cytokine responses, intestinal injury, and disease severity, and deletion of Glp1r in donor cells did not materially alter the major outcomes [202]. These negative findings caution against directly extrapolating the anti-inflammatory and barrier-protective effects reported in metabolic or sterile intestinal injury to transplantation-associated immune pathology.

Interpretation in high-risk hosts requires a clear distinction among the risk of acquiring infection, the likelihood of severe disease, and the response to treatment after infection has occurred [14, 160]. Sustained GLP-1RA exposure may reduce infection susceptibility or progression to severe disease through improvements in glycaemia, weight, kidney function, cardiovascular health, and overall physiological reserve. Such effects represent optimisation of baseline host risk and tolerance rather than direct modification of pathogen replication or clearance during established infection [41, 161, 203]. During acute infection, vomiting, poor intake, dehydration, haemodynamic instability, and fluctuating kidney function may alter both GLP-1RA tolerability and the appropriateness of continued treatment. Pre-existing long-term exposure, continuation during acute illness, and de novo initiation after diagnosis should therefore be treated as distinct clinical questions [204, 205].

Clinical evidence from observational cohorts, trial-level syntheses, and secondary randomised analyses in patients with diabetes, obesity, chronic kidney disease, or cardiovascular disease consistently associates pre-existing GLP-1RA exposure with fewer infections or less severe outcomes [14, 41, 160, 161]. The available findings are most consistent with long-term metabolic optimisation, cardiorenal protection, and preservation of organ reserve, while infection-specific evidence in iatrogenic immunosuppression and haematopoietic stem-cell transplantation is sparse [200–202]. Further studies require stratification by glycaemic control, obesity severity, kidney function, cardiovascular reserve, and the intensity of immunosuppression. Randomised trials initiating treatment after infection will ultimately be required to define the therapeutic role of GLP-1RAs across distinct high-risk host populations (Table 3).

**Table 3:** Clinical evidence on GLP-1RA exposure and infection-related outcomes

Evidence type/design	Exposure/population	Infection outcome and result	Interpretation	Reference(s)
SELECT secondary analysis	Randomised semaglutide 2.4 mg weekly vs placebo; adults with overweight/obesity and cardiovascular disease	COVID-19 incidence was not materially reduced, but serious/fatal COVID-19 outcomes were fewer	Randomised chronic exposure supports a severity signal; it does not test treatment started after infection diagnosis	[160]
FLOW infection analysis	Randomised semaglutide 1.0 mg weekly vs placebo; type 2 diabetes with chronic kidney disease	Serious infections and infection-related hospitalisation were less frequent	High-risk CKD population; infection was not the primary treatment indication and exposure preceded infection	[161]
N3C observational cohort	Premorbid GLP-1RA use vs adjusted DPP-4 inhibitor use in adults with type 2 diabetes and COVID-19	Lower adjusted 60-day mortality and selected severe outcomes	Residual confounding remains; the study does not establish benefit from de novo acute treatment	[158]
Active-comparator real-world cohort	GLP-1RA vs DPP-4 inhibitor initiators with 12-month follow-up	Lower adjusted risks of incident pneumonia and severe sepsis	Association may reflect metabolic control, healthcare behaviour and residual confounding	[14]
COVID-19 systematic review/meta-analysis	Observational studies of preadmission or prediagnosis GLP-1RA exposure in diabetes	Pooled estimates favoured prior exposure for COVID-19 mortality	Heterogeneous observational evidence; no inference about treatment initiated during acute infection	[150]
Meta-analysis of randomised trials	Chronic GLP-1RA exposure in metabolic, cardiovascular, renal and obesity trials	Lower risk of serious infections; organ-specific signals varied	Trials were not designed to test infection treatment, pathogen clearance or post-diagnosis initiation	[41]

This table summarises selected clinical evidence discussed in the review and does not represent a systematic evidence search. Most studies concern chronic exposure before infection or secondary or post hoc infection analyses rather than de novo GLP-1RA initiation during acute infection

*Abbreviations*: *CKD* chronic kidney disease, *COVID-19* coronavirus disease 2019, *DPP-4* dipeptidyl peptidase-4, *GLP-1RA* glucagon-like peptide-1 receptor agonist, *N3C* National COVID Cohort Collaborative

## Therapeutic translation: pharmacology, safety, and clinical context

Having reviewed the mechanistic and disease-specific evidence, the next challenge is to determine how these biological effects might translate into clinically meaningful use. Such translation depends not only on whether GLP-1RAs influence infection-related host responses, but also on how agent-specific pharmacology, treatment timing, host characteristics, concomitant therapies, and acute-phase safety shape the balance between potential benefit and risk. These considerations are particularly important because chronic exposure before infection is biologically and clinically distinct from de novo treatment initiated during acute illness, and because GLP-1RAs differ substantially in pharmacokinetics, tissue exposure, receptor signalling, and tolerability. The translational relevance of GLP-1RAs in infectious disease should therefore be evaluated within the specific pharmacological and clinical context in which treatment is used.

### Pharmacological differences among GLP-1RAs

Currently available GLP-1RAs should not be regarded as a pharmacologically homogeneous drug class. Individual agents differ substantially in molecular design, pharmacokinetic duration, dosing schedule, tissue distribution, receptor-binding kinetics, and biased signalling profiles. Assessment of GLP-1RAs in infection should therefore be agent specific and should integrate pharmacokinetics, tissue exposure, receptor-signalling properties, and the biological requirements of the particular infectious setting [206].

Exenatide, an exendin-4-derived peptide, was one of the earliest GLP-1RAs to enter clinical use. Short-acting exenatide has a terminal half-life of approximately 2.4 h and requires twice-daily subcutaneous administration, producing pronounced peak-to-trough exposure that is particularly suited to studies of postprandial glycaemia, gastric emptying, and acute metabolic responses [207]. The biodegradable microsphere formulation exenatide LAR permits once-weekly administration and produces a gradual rise in systemic exposure [208, 209]. Steady state is typically reached after approximately 6–7 weeks, although population pharmacokinetic modelling suggests that 8–10 weeks may be required in some settings. These kinetics are better suited to studies of sustained metabolic and chronic inflammatory effects [208, 209].

Liraglutide is a human GLP-1 analogue modified with a C16 fatty acid side chain that promotes reversible albumin binding, slows absorption, and limits renal clearance. Relative to short-acting exenatide, liraglutide provides more stable daily exposure and is thus more suitable for investigating sustained metabolic, vascular, anti-inflammatory, and organ-protective effects, although its duration remains shorter than that of weekly dulaglutide or semaglutide [210, 211].

Dulaglutide is an Fc-fusion macromolecule comprising modified GLP-1 analogue chains linked to a human IgG4 Fc fragment, thereby reducing renal elimination and extending circulatory persistence. After subcutaneous dosing, dulaglutide reaches peak concentration at approximately 48 h, has a terminal half-life of approximately 5 days, and achieves steady state between the second and fourth doses, enabling once-weekly administration [212, 213]. As an Fc-fusion macromolecule, dulaglutide may differ from smaller peptide agonists in tissue distribution and local receptor exposure. These factors should therefore be considered when interpreting tissue-specific infection-model outcomes.

Semaglutide combines amino acid substitution with a C18 fatty acid side chain, resulting in high-affinity albumin binding and resistance to DPP-4 degradation. The approximately 7-day half-life of subcutaneous semaglutide permits once-weekly administration, with steady state typically achieved after 4–5 weeks [214]. Oral semaglutide uses the absorption enhancer sodium N-(8-[2-hydroxybenzoyl]amino) caprylate (SNAC) to facilitate gastric absorption and differs from the subcutaneous formulation in bioavailability, absorption kinetics, administration conditions, and systemic exposure, making formulation-specific pharmacokinetic considerations essential in infection research [215].

Tirzepatide is a long-acting acylated dual agonist of the glucose-dependent insulinotropic polypeptide receptor (GIPR) and GLP-1R, with an approximately 5-day half-life that supports once-weekly dosing [216, 217]. Its biological effects therefore cannot be assigned to GLP-1R alone [216, 218]. Infection studies of tirzepatide must separate GIPR-mediated metabolic, adipose, and potential immune effects from GLP-1R-dependent actions and should not extrapolate dual-agonist outcomes to GLP-1R-specific mechanisms [216, 218].

Tissue exposure is also agent dependent, because molecular size, albumin binding, Fc fusion, depot formulation, and clearance pathways can generate distinct distribution profiles across pancreatic, gastrointestinal, pulmonary, cardiovascular, renal, neural, and immune compartments. This distinction is particularly relevant to infection because GLP-1RA-associated protection may arise through coordinated metabolic, neuroimmune, vascular, and multiorgan barrier effects rather than through a single local receptor action [5, 8].

Although exenatide, liraglutide, dulaglutide, and semaglutide all engage the canonical GLP-1R–Gαs–cAMP–PKA/Epac pathway, differences in ligand architecture, binding kinetics, β-arrestin recruitment, receptor trafficking, and signal persistence may produce distinct patterns of desensitisation and downstream biological activity [219, 220]. Tirzepatide exhibits an intrinsically more complex signalling profile because its unequal and potentially biased activation of both GIPR and GLP-1R cannot be accommodated within a single-receptor model of GLP-1R-biased agonism [218].

Collectively, pharmacokinetic duration, tissue distribution, and receptor-signalling properties determine the experimental and potentially clinical contexts in which individual GLP-1RAs can be meaningfully evaluated. Short-acting agents are most informative for acute metabolic, gastrointestinal, and early inflammatory responses, whereas long-acting formulations are better suited to sustained metabolic and organ-protective endpoints, and tirzepatide requires explicit separation of its GIPR- and GLP-1R-mediated components. Future infection studies should match the individual agonist, formulation, dosing schedule, treatment timing, pathogen context, and disease stage and should not assume class-wide interchangeability (Table 4).

**Table 4:** Pharmacological features of incretin-based agents relevant to infection research

Agent/class	Typical exposure/pharmacology	Infection evidence and acute-phase implication	Reference(s)
Exenatide	Short-acting; usually twice-daily s.c.; predominantly renal elimination	Direct infection-intervention evidence is sparse. Short exposure aids temporal control, but renal dysfunction and dehydration may increase exposure or tolerability concerns	[221, 222]
Liraglutide	Once-daily s.c.; albumin binding; half-life approximately 13 h	Most diverse preclinical infection evidence, including bacterial pneumonia, sepsis, influenza and malaria. Infection-after-diagnosis efficacy remains model-dependent	[13, 147–149, 211]
Dulaglutide	Once-weekly s.c.; GLP-1 analogue–Fc fusion; prolonged exposure	One LPS-induced acute lung injury study supports lung anti-inflammatory effects, but no live-pathogen efficacy is established in the current evidence set. Long exposure limits rapid withdrawal	[11, 213]
Semaglutide	Once-weekly s.c. or once-daily oral; systemic half-life approximately 1 week	SELECT and FLOW provide chronic pre-infection exposure signals rather than acute-treatment evidence. Persistent exposure and gastrointestinal effects are relevant during acute illness	[160, 161, 214]
Tirzepatide	Once-weekly dual GIPR/GLP-1R agonist; half-life approximately 5 days	Direct infection-focused evidence is insufficient. Observed effects cannot be assigned to GLP-1R alone without receptor-selective controls	[217, 218]

The evidence summary is limited to studies discussed or cited in the current review and is not a systematic-search claim. Abbreviations: *Fc* fragment crystallisable, *GIPR* glucose-dependent insulinotropic polypeptide receptor, *GLP-1R* glucagon-like peptide-1 receptor, *LPS* lipopolysaccharide, *s.c.* subcutaneous

### Combination strategies and safety

In infectious diseases, GLP-1RAs should not be considered substitutes for antimicrobial or antiviral therapy, source control, haemodynamic resuscitation, or organ support, but rather as candidate adjuncts that may improve metabolic–inflammatory homeostasis, barrier integrity, and organ tolerance [4, 24]. Infection outcomes reflect the combined effects of pathogen burden, immune competence, baseline metabolic health, and physiological organ reserve. Their translational value should be judged by whether they can complement standard anti-infective care by limiting metabolic stress, immunopathology, and secondary organ failure without impairing pathogen elimination, rather than by direct antimicrobial activity [8, 223, 224].

GLP-1RAs and SGLT2 inhibitors are widely combined in chronic cardiometabolic care because their effects on glycaemia, body weight, and cardiorenal protection are partly complementary [225]. Infection-related evidence, however, is based largely on chronic-exposure cohorts, secondary trial endpoints, and real-world analyses rather than on prospectively designed combination studies during acute infection [226]. During acute illness, fasting, hypovolaemia, or insulin deficiency, SGLT2 inhibitors increase the risk of euglycaemic ketoacidosis, while GLP-1RA-related gastrointestinal adverse effects may compound dehydration, inadequate nutrition, and acute kidney injury [227–229]. The chronic benefits of this combination should therefore not be extrapolated to acute infection or critical illness, in which haemodynamics, nutritional intake, volume status, renal function, and ketoacidosis risk should guide treatment decisions.

The rationale for combining GLP-1RAs with antibiotics is host optimisation rather than pharmacological enhancement of antibiotic killing. Improvements in glycaemic control and inflammatory regulation, together with organ-protective effects reported in preclinical models, may provide a more favourable host environment during infection [13, 61, 176]. No adequate evidence currently shows that GLP-1RAs potentiate specific antibiotics, shorten treatment courses, or improve major sepsis outcomes [24]. GLP-1RA-induced slowing of gastric emptying may also delay or alter oral drug exposure, which is particularly relevant when rapid and predictable antimicrobial concentrations are required during severe infection or intravenous-to-oral step-down therapy [178].

In viral disease, GLP-1RAs would similarly serve as host-directed adjuncts rather than as direct antiviral agents. Observational COVID-19 studies and secondary analyses of large cardiometabolic and renal trials associate chronic GLP-1RA exposure, including semaglutide, with fewer severe infections, COVID-19-related adverse events, or infection-related deaths [41, 160, 161]. During acute viral infection, fever, reduced intake, and dehydration may amplify GLP-1RA-related gastrointestinal intolerance and alter both nutritional status and oral antiviral exposure. These associations neither establish inhibition of viral replication nor justify replacement of pathogen-directed antiviral therapy.

Combining GLP-1RAs with glucocorticoids, IL-6 or JAK inhibitors, or complement-targeted therapies requires particular vigilance for cumulative immunosuppression. Although multitarget immunomodulation may further restrain inflammatory amplification and endothelial injury, effective pathogen control still requires preserved innate and adaptive immune function [88, 223]. Additional inflammatory suppression may be harmful during uncontrolled pathogen replication, before effective anti-infective therapy is established, or in patients with lymphopenia and secondary immunosuppression because it may delay clearance and increase secondary infection risk [24]. Combination immunomodulation should not be guided by isolated cytokine reductions alone; it should be considered only in the presence of adequate pathogen-directed therapy, measurable infection control, and an acceptable immunosuppressive risk profile.

Pharmacokinetic controllability and reversibility remain important safety considerations during acute infection, particularly for long-acting GLP-1RAs in patients with haemodynamic instability, renal dysfunction, gastrointestinal intolerance, or poor nutritional intake [110, 228]. Because chronic pre-infection use, inpatient continuation, temporary interruption, and de novo initiation represent distinct clinical scenarios, their detailed clinical evaluation is addressed in the section on clinical translation.

Future development should prioritise infection-relevant pharmacology rather than simply maximising glycaemic efficacy, weight loss, or duration of action. GLP-1R ligands differentially engage Gαs–cAMP–PKA/Epac signalling, β-arrestin recruitment, receptor trafficking, and sustained endosomal signalling, producing distinct biased-signalling profiles [5, 219, 230]. An ideal infection-oriented candidate should preserve metabolic stability, barrier integrity, and organ tolerance without compromising phagocyte-mediated killing, interferon responses, or effector T-cell function. Tissue-selective targeting, local administration, and inflammation-responsive delivery may theoretically increase exposure within target organs while limiting systemic drug exposure. To date, infection-related targeted-delivery research has focused mainly on preclinical nanoplatforms for sepsis and acute lung injury, whereas infection-specific GLP-1R-targeted formulations remain unavailable and the relevant technologies are still largely at the proof-of-concept or early preclinical stage [231–233].

Overall, combinations of GLP-1RAs with metabolic, anti-infective, or immunomodulatory therapies should be regarded as investigational adjunctive strategies rather than established standards of infection care. Drug development should preserve metabolic and organ-protective benefits while improving controllability during acute illness, with maintenance of pathogen clearance and protective immunity regarded as indispensable safety requirements.

### Clinical translation: integrating pharmacology, host context, and treatment setting

Clinical translation of GLP-1RAs in infectious disease is unlikely to be determined by a single pharmacological or immunological effect. Rather, their clinical relevance is likely to depend on the interaction between agent-specific pharmacology, host phenotype, infection stage, and treatment setting. Differences in molecular design, pharmacokinetics, tissue exposure, and receptor signalling mean that findings obtained with one GLP-1RA cannot automatically be extrapolated across the class. In parallel, the safety and combination considerations indicate that the consequences of GLP-1RA exposure may change substantially during acute infection, when haemodynamic instability, altered nutritional intake, metabolic disturbances, and organ dysfunction may modify both drug tolerance and therapeutic priorities. The key translational question is therefore not simply whether GLP-1RAs are beneficial during infection, but which agent is appropriate for which host, at what stage of disease, and for what therapeutic objective [206, 219].

This distinction is particularly important when interpreting the available human evidence. Observational studies, real-world analyses, and secondary analyses of cardiometabolic or renal trials have associated chronic GLP-1RA exposure with lower risks of selected infections or severe infection-related outcomes in some populations [14, 160, 161]**.** However, these findings predominantly reflect treatment established before infection onset and therefore incorporate the cumulative effects of chronic metabolic, vascular, and cardiorenal modification. They should not be assumed to predict the effects of de novo GLP-1RA initiation after infection has developed, when inflammatory, haemodynamic, metabolic, and organ-level disturbances may already be established. Clinically, established users and treatment-naïve patients represent distinct translational setting**s**: in the former, the question concerns continuation, temporary interruption, or resumption of an existing therapy, whereas in the latter, GLP-1RA administration represents a new intervention introduced during an evolving acute illness. These two clinical scenarios should be evaluated separately.

Host phenotype provides a further level of clinical stratification. GLP-1RA therapy may be particularly relevant when metabolic dysregulation, obesity-related inflammation, endothelial dysfunction, or cardiorenal vulnerability contributes substantially to infection-associated physiological stress, because these abnormalities overlap with domains in which GLP-1RAs have established systemic effects. Severe physiological instability, advanced organ dysfunction, or profound immune impairment may alter treatment priorities [234, 235], and substantial gastrointestinal intolerance may further limit GLP-1RA use during acute illness [110]. Diabetes or obesity alone may therefore be insufficient to define the clinically relevant population.

A more informative framework would integrate metabolic reserve, cardiorenal function, immune competence, endothelial or barrier integrity, and acute disease severity, allowing treatment effects to be interpreted in relation to the broader host context rather than a single diagnostic category.

The therapeutic objective must also remain clearly defined. GLP-1RAs are not substitutes for pathogen-directed antimicrobial therapy, and their potential value in infection is more plausibly related to modulation of the host response, including attenuation of metabolic stress, preservation of endothelial and barrier function, and reduction of organ vulnerability. Accordingly, translational success should not be judged solely by reductions in inflammatory biomarkers, but by whether host protection translates into clinically meaningful preservation of organ function or recovery without compromising pathogen control or protective immunity. Within this framework, GLP-1RAs are best considered candidate host-directed adjuncts in selected clinical settings, with their potential use determined by the alignment of agent, host phenotype, disease stage, and treatment context (Fig. 2b).

## Conclusions and outlook

### Integrated conclusions and conceptual framework

Current evidence supports the GLP-1/GLP-1R axis as a context-dependent regulator of infection-associated host responses, rather than a uniformly anti-inflammatory or directly antimicrobial pathway. Its effects appear to arise through an integrated network involving systemic metabolic regulation, central neuroimmune signalling, peripheral barrier and organ protection, and, in selected settings, potentially cell-specific receptor actions. Across the evidence reviewed here, metabolic and neuroimmune effects are more consistently supported, while direct receptor-dependent actions in many immune-cell populations remain incompletely established. Treatment-associated changes in inflammatory or immune phenotypes cannot therefore be assumed to reflect immune-cell-intrinsic GLP-1R signalling.

Across the studies reviewed here, GLP-1R-related interventions more consistently attenuate inflammatory injury, preserve tissue integrity, and improve organ-related outcomes than enhance pathogen elimination. This pattern is more consistent with host tolerance than with a general increase in host resistance. Both processes may be affected in selected settings, but reduced inflammation or tissue injury alone does not establish improved antimicrobial defence. Infection-related outcomes are therefore best considered by separating host resistance from host tolerance and direct receptor-dependent actions from indirect systemic effects.

Clinical interpretation also depends on distinguishing chronic pre-infection exposure from treatment initiated after infection has become established. Findings should be considered in relation to the specific agent, host context, and disease setting rather than assumed to represent a uniform class effect. Four complementary distinctions help organise this evidence: host resistance versus host tolerance, direct receptor-dependent actions versus indirect systemic effects, chronic host conditioning versus acute pharmacological intervention, and agent-specific effects versus assumed class-wide effects. These distinctions provide a framework for interpreting both the current evidence and its translational implications.

### Major limitations of the current evidence

Several limitations currently constrain mechanistic interpretation and clinical translation of GLP-1/GLP-1R signalling in infection. A fundamental challenge is the uncertain cellular assignment of functional GLP-1R. Methodological limitations in receptor detection and functional validation continue to complicate reliable assignment of GLP-1R to specific immune-cell populations. As a result, direct receptor-dependent effects remain difficult to distinguish from indirect systemic responses.

Experimental relevance is also limited because much of the mechanistic evidence reviewed here derives from LPS endotoxaemia, sterile inflammation, metabolic disease, colitis, or acute tissue-injury models rather than defined live-pathogen infections. Although these models are useful for dissecting inflammatory signalling and tissue injury, they do not fully reproduce pathogen replication, antimicrobial exposure, evolving host immunity, or pathogen clearance. Findings from such surrogate models therefore cannot independently establish infection-specific therapeutic efficacy.

Another limitation is the incomplete integration of pathogen-related and host-related outcomes. Across the studies reviewed here, inflammatory, histological, barrier, and organ-related outcomes are often assessed without parallel measures of microbial burden or pathogen clearance. Reduced inflammation or improved tissue integrity may therefore indicate enhanced host tolerance without demonstrating improved host resistance, whereas tissue protection cannot automatically be considered beneficial if protective antimicrobial responses are impaired.

Finally, pharmacological and clinical heterogeneity limits extrapolation across studies. Differences in agent, treatment timing, host phenotype, disease severity, and concomitant therapy complicate comparison across experimental and clinical settings. The human evidence reviewed here predominantly reflects chronic pre-infection exposure, leaving the effects of treatment initiated during established infection insufficiently defined. Together, these limitations identify the main methodological and translational gaps that future studies need to address.

### Priorities for future research and translation

Addressing these limitations will require a more rigorous and context-sensitive research framework centred on receptor validation, infection-relevant experimental models, parallel assessment of pathogen and host outcomes, and clinically meaningful treatment settings.

A first priority is to establish the cellular sites at which functional GLP-1R signalling contributes to infection-related responses. This will require orthogonal receptor-validation approaches that combine validated receptor detection, ligand-binding and functional signalling assays, and cell-specific genetic perturbation [33]. Where immune-cell-intrinsic mechanisms are proposed, metabolically matched comparator groups should be incorporated to separate direct receptor-dependent effects from secondary consequences of altered glycaemia, body weight, or systemic metabolism.

A second priority is to strengthen the infection relevance of experimental models. Future studies should prioritise defined live-pathogen and post-infection treatment designs incorporating clinically relevant disease stage, antimicrobial therapy, and host background, so as to determine whether mechanisms identified in sterile or metabolic settings remain operative during active infection.

Future studies should also assess pathogen control and host protection in parallel. Pathogen burden and clearance kinetics should be evaluated alongside immune function, tissue injury, organ dysfunction, and survival, allowing enhanced host resistance to be distinguished from improved host tolerance [67]. Reduced inflammation or tissue injury alone should not be interpreted as evidence of improved antimicrobial defence unless pathogen control is preserved.

Clinical translation further requires clearer definition of treatment timing, patient selection, and acute-phase safety. Chronic pre-infection exposure and de novo treatment after infection onset should be evaluated separately. Future studies should account for infection severity, metabolic and immune status, organ function, concomitant therapy, and agent-specific pharmacology, and should determine whether GLP-1R modulation can provide meaningful host protection without compromising pathogen clearance or protective immunity.

Overall, the available evidence positions GLP-1/GLP-1R signalling as a context-dependent modifier of infection-associated host responses, with more consistent evidence for metabolic regulation, neuroimmune control, barrier preservation, and organ protection than for direct enhancement of pathogen clearance. Important uncertainties remain regarding cell-specific receptor function, infection-relevant experimental models, and the distinction between host resistance and host tolerance. Resolving these questions will require receptor-specific validation, post-infection live-pathogen studies, integrated pathogen and host outcome assessment, and clinically relevant evaluation of timing and safety. Until such evidence is available, GLP-1RAs should be regarded as candidate host-directed adjuncts rather than established anti-infective therapies.

## Data Availability

Not applicable.
